# Mycorrhizal symbiosis maintains DNA integrity and cellular homeostasis in lettuce under combined heavy metals and salt stress conditions

**DOI:** 10.1186/s12870-026-09544-0

**Published:** 2026-08-08

**Authors:** Rabab A. Metwally, Elham R. S. Soliman

**Affiliations:** 1 Botany and Microbiology Department, Faculty of Science, Zagazig University, Zagazig, Egypt; 2 Botany and Microbiology Department, Faculty of Science, Capital University (Formerly Helwan University), Cairo, Egypt

**Keywords:** Antioxidant enzymes, Genome profiling, Glomalin, Metal uptake, Mycorrhiza, Lettuce, Phosphatases, Single cell gel electrophoresis

## Abstract

**Background:**

Combination stresses are the primary obstacle that plants encounter in nature. Consequently, there is an urgent need for environmentally sustainable solutions. Arbuscular mycorrhizal fungi (AMF) constitute one such eco-friendly approach for promoting agricultural sustainability under heavy metals (HMs) contamination, drought, heat, and salinity stress conditions, owing to their well-documented role as biostimulants. Therefore, the present investigation aimed to assess the impact of combined HMs (Cr, Pb, and Cd; each at 100 mg L^− 1^) and salt stress (100 mM NaCl) on lettuce and to evaluate the potential of AMF inoculation to mitigate these combined stresses.

**Results:**

AMF colonization effectively mitigated this adverse effect, as evidenced by increases in shoot fresh weight (12.97%), total pigment (76.74%), relative water content (RWC, 10.98%), glycine betaine (GB, 21.04%), and phenolic content (15.68%). Conversely, a substantial decrease in stress markers was observed, including lipid peroxidation (MDA, 11.03%), H_2_O_2_ content (20.66%), and reductions in the antioxidant enzymes (23.56% in POD, 22.38% in PPO, and 14.73% in phenylalanine ammonia-lyase [PAL]). The Start codon targeted (SCoT) analysis demonstrated that all of these were associated with a 51% retention of genome integrity, and the percentage of damaged nuclei was reduced by 25.78%, as confirmed by the comet assay. Both easily extractable (EE, 29.58%) and total extractable (TE, 36.42%) glomalin content were significantly increased by AMF colonization upon stress. Although AMF-colonized roots exhibited a higher concentration of Pb, Cr, and Cd during combined stress, the concentration of these metals in the shoots was lower. This reduction was attributed to the ability of AMF to reduce the root-shoot translocation by 74.39, 61.42, and 56.92% for Cr, Pb, and Cd, respectively.

**Conclusion:**

This method could be used to cultivate lettuce in contaminated locations by trapping HMs and Na^+^ ions in the roots, which are not edible, while allowing the edible shoots to develop. Thus, AMF could be used to protect food safety and agricultural output in contaminated areas.

**Graphical abstract:**

**Figure:**
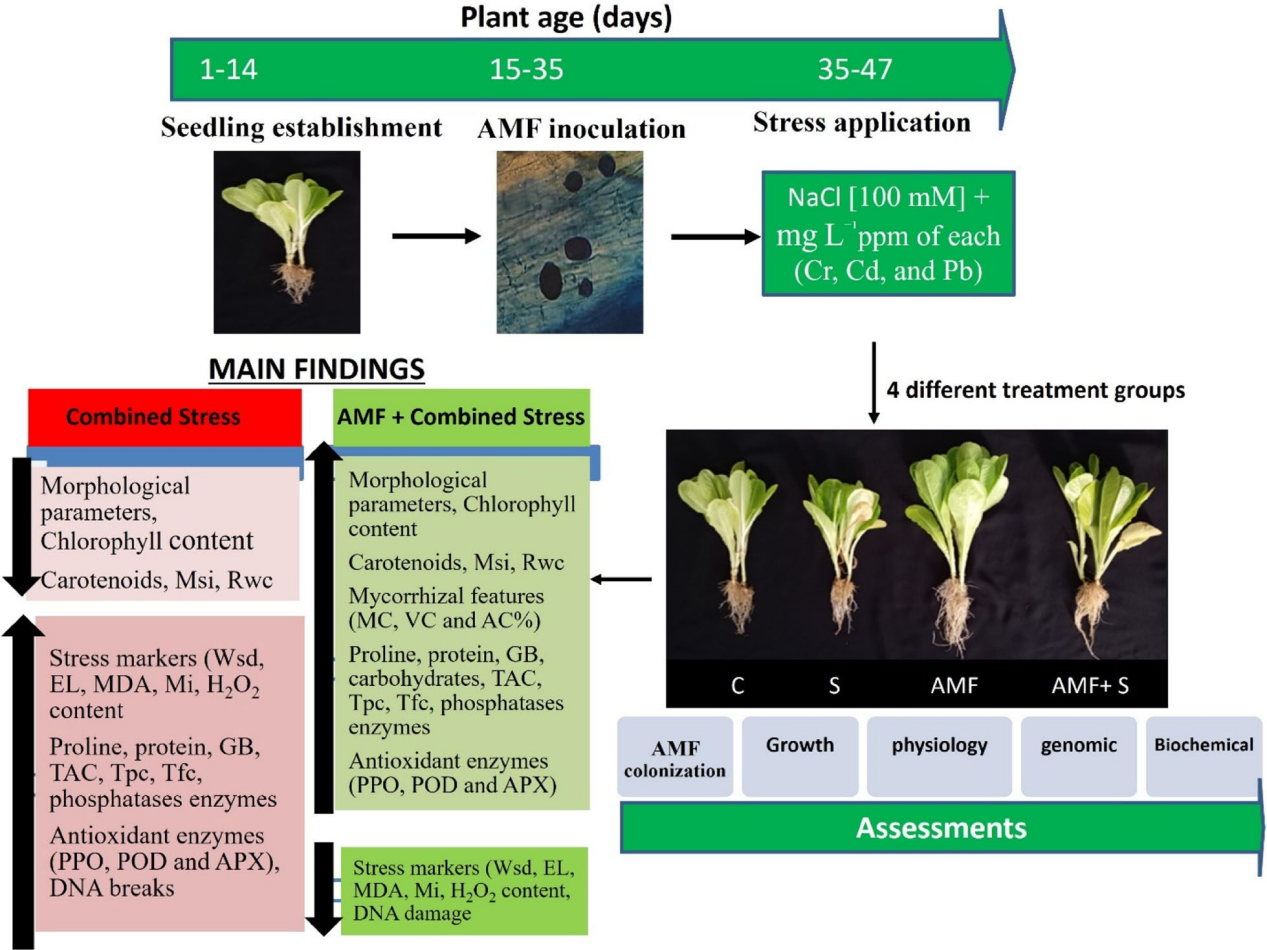
Experimental layout and the main findings of the study. Lettuce (*L. sativa* L.) seedlings were AMF-inoculated, grown for 20 days after transplanting, and then subjected for 12 days to the combined HMs (100 mg L^− 1^ of each Pb, Cr, and Cd) and salt (100 mM NaCl) stress. Increase is indicated with an arrow with the top pointing up, and decrease is indicated with a downward-pointing arrow. *C; refers to control, S; stressed plants with 100 mg L^− 1^ of Cr, Cd, and Pb and 100 mM NaCl, AMF; AMF-inoculated plants, and AMF + S; AMF-inoculated and stressed with a combination of 100 mg L^− 1^ of Cr, Cd, and Pb and 100 mM NaCl

**Supplementary Information:**

The online version contains supplementary material available at 10.1186/s12870-026-09544-0.

## Introduction

Lettuce (*Lactuca sativa* L.) is one of the most extensively cultivated leafy vegetables worldwide, with an annual production of approximately 28 million tons [1]. It is a valuable source of bioactive compounds, including polyphenols, chlorophyll, and carotenoids, and contains nutraceutical and phytochemical constituents with documented anticarcinogenic properties [2]. As a leafy, fast-growing crop, lettuce is particularly vulnerable to soil pollution and functions as a direct pathway through which contaminants such as heavy metals (HMs) and salts enter the human food chain. For example, lettuce cultivated under Cd contamination exhibited significantly reduced dry mass and abnormal, truncated, yellow morphology, accompanied by elevated antioxidant enzyme activity, total soluble protein, and total phenolic content as part of the plant’s defense response [3].

In agroecosystems, the co-occurrence of HMs and salinity stress is increasingly common. Growing pressure on freshwater resources, driven by population growth, agricultural expansion, and climate variability, has increased reliance on alternative irrigation sources such as brackish water, treated wastewater, and agricultural drainage water [4]. While these sources help offset water scarcity, they frequently carry elevated salt concentrations and potentially toxic HMs, contributing to soil salinization and combined stress in plants [5]. Certain HMs (e.g., Cu, Ni, Zn) are essential micronutrients, but their excess accumulation, along with that of nonessential HMs such as Cr, Pb, and Cd, is toxic to plants, and this toxicity can be intensified by antagonistic or synergistic interactions among co-occurring HMs [6, 7].

When HMs and salinity stresses occur together, their interaction substantially alters plant growth and metal accumulation dynamics relative to either stress alone, driving the overproduction of reactive oxygen species (ROS) that damage DNA, proteins, and lipids [8, 9]. Salinity, in particular, influences the mobility, bioavailability, and uptake of HMs in plants, though this relationship is highly dependent on plant species, metal type, and environmental conditions [10]. For instance, Hossain et al. [11] reported increased Cd, Zn, and Cu accumulation in maize under NaCl and NaCl + CaCl_2_ treatments, attributed to enhanced metal solubility and altered membrane permeability under saline conditions. Aryal [12] similarly noted that metal uptake depends strongly on the concentration and interaction of metal ions with sodium salts. Species- and metal-specific responses have also been documented: Schück et al. [13] found that *Phalaris arundinacea* most effectively removed Cd, Zn, and Cl⁻, while *Carex pseudocyperus* was more effective for Cu and Pb, with increasing salinity reducing Cd and Pb removal but not affecting Zn and Cu uptake. Comparable variability has been reported in *Kosteletzkya pentacarpos* [14], *Beta vulgaris* [15], *Solanum tuberosum* [16], and *Zea mays* [17], with sensitivity also differing between crops such as rocket (more salt-sensitive) and parsley (more HMs-sensitive) [18].

Our study is particularly relevant to real-world contamination scenarios, as the concentrations selected (100 mM NaCl; 100 mg L⁻¹ each of Cr, Pb, and Cd) reflect levels reported in industrial discharge areas and heavily polluted agricultural environments. These three metals were specifically selected because they frequently co-occur at contaminated sites and exhibit distinct toxicity mechanisms in plant systems [19–21], while the NaCl concentration used corresponds to levels commonly recorded in brackish water systems and saline-affected soils [22].

Arbuscular mycorrhizal fungi (AMF) form a widespread mutualistic symbiosis with plant roots, in which an extensive extraradical hyphal network enhances plant acquisition of phosphorus, nitrogen, and micronutrients such as Zn and Cu, in exchange for photosynthetically derived carbon from the host [23, 24]. Because of this capacity, AMF represent a natural, environmentally sustainable means of alleviating both salinity and HMs stress through coordinated molecular and physiological mechanisms [25, 26]. In *Acacia adsurgens*, for example, AMF inoculation improved the shoot K⁺: Na⁺ ratio and P uptake while reducing Na⁺ and Cd concentrations and translocation, with transcriptome analysis linking the resulting biomass gains to AMF-mediated upregulation of cytokinin-related genes [27]. More broadly, AMF can mitigate combined NaCl and HMs stress by modulating cell growth, redox balance, cell wall assembly, and associated biochemical and genetic pathways [26, 28].

Despite this, the protective role of AMF against the simultaneous occurrence of HMs toxicity and salinity stress in lettuce remains largely unexplored. To the best of our knowledge, this is the first study to investigate the combined effects of multiple HMs (Cr, Pb, and Cd; each at 100 mg L⁻¹) together with 100 mM NaCl on lettuce. Moreover, most prior work on AMF-mediated stress tolerance has focused on physiological and biochemical responses, leaving genomic and DNA-protective mechanisms comparatively underexplored. The present study, therefore, offers an integrated assessment of AMF-induced mitigation of combined HMs-salinity stress, combining growth performance, physio-biochemical traits, antioxidant defense, and DNA/genomic stability (via comet assay, ISSR, and SCoT markers) to provide deeper mechanistic insight into AMF-mediated stress tolerance in lettuce.

## Materials and Methods

### Arbuscular Mycorrhizal Fungal (AMF) inoculum

Using wet sieving and decanting techniques [29], the spores of *Funneliformis mosseae*, *F. constrictum*, *Gigaspora margarita*, and *Rhizophagus irregularis* were isolated from El-Sharkia Governorate soil. They were then propagated in trap culture (culture medium of autoclaved sand-clay soil (1:1, w/w)) using Sudan grass (*Sorghum sudanense* Pers.). Inoculum potential of AMF was measured by the number of spores (23 spore/g) and the percentage of mycorrhizal colonization (MC) of the roots [29, 30]. Microscopic examination revealed near-complete colonization of the roots by hyphae, vesicles, and arbuscules.

### Heavy Metals (HMs) and salt stress treatment

Cr, Pb, and Cd were applied to the irrigation water as metallic salt solutions at a rate of 100 mg L^− 1^ for each one as K_2_Cr_2_O_7_, Pb (C_2_H_3_O_2_)_2_, and CdCl_2_, respectively. HMs salts were used in a chemical pure grade (Merck). As well, a salt solution of one level (100 mM) of NaCl was prepared and applied to the irrigation water. Tap water was used to irrigate the control treatments.

### Experimental design and growth conditions

Lettuce (*Lactuca sativa* L.) (Lettuce Nader, US, GP 95%) seedlings of 2 weeks old were used as plant material and obtained from a nursery greenhouse of seedling development from Al-Maymounah Village, Minya Al Qamh, Al-Sharqia Governorate. A factorial arrangement on a randomized complete block design was conducted in a research greenhouse of the Faculty of Science, University of Zagazig, Egypt, during the autumn and winter seasons of 2024–2025 (natural light, 21 ± 2 °C during the daytime while, 12 ± 2 °C at night, with an 11-h light and 13-h dark photocycle, and the relative humidity is ranged (60–70%). Lettuce seedlings were transplanted in plastic pots (3 seedlings/pot) containing 3 kg of sterilized clay soil [(62.8% clay, 25.5% silt, and 9.7% sand), pH of 7.7, electric conductivity of 1.93 dS m^− 1^, saturation percentage of 60%, anion content of SO_4_^2−^ = 6.57 and HCO_3_^−^ = 5.66, and cation content of K^+^ = 0.42, Mg^2+^ = 4.98, and Ca^2+^ = 10.61 mEq L^− 1^]. AMF inoculum was added to mycorrhiza-treated seedlings below their roots during transplanting as 25 g of AMF inoculum/pot that contained spores (23 spore/g), hyphae, colonized root fragments (≈ 100%, colonization index), and soil. The same amount of autoclaved AMF inoculum and the AMF inoculum filtrate (filtered with a pore size of < 20 μm) were added to the non-AMF pots to restore a native microbial community free of AMF propagules. All pots were irrigated well with tap water for 20 days.

Our study is pertinent to comprehending the potential consequences of combined HMs-salinity stress in real-world scenarios, as the concentrations selected (100 mM NaCl, 100 mg L^− 1^ HMs) are indicative of contamination scenarios that may arise in industrial discharge areas and highly polluted environments. Therefore, the combined HMs-saline [(Cr, Pb, and Cd) each at a concentration of 100 mg L^− 1^ in conjunction with 100 mM NaCl] was used as a stress treatment and initiated 20 days after transplantation and AMF inoculation.

Therefore, the study consisted of four treatments, outlined in Table 1. The experiment was accomplished with a randomized complete block design with five replicas (pots) for each treatment (4 × 5). Each replica enclosed 3 similar-looking seedlings. The pots were irrigated regularly with water or stress solutions for 12 days till the appearance of stress disorders. Each pot was irrigated at a 3-day interval with 250 mL of either tap water (for no-stress treatment groups) or otherwise HMs-saline (for stress treatment groups). Collectively, each pot of the S and AMF + S groups received a total of 1000 mL of HMs-saline solution for the experimental duration.

**Table 1:** The in vivo experimental treatment groups of the study

Treatments and their code	Explanation
Control (C)	The lettuce seedlings without stress or AMF inoculation.
Mycorrhizal (AMF)	The lettuce seedlings were supplemented with AMF inoculum.
Stressed (S)	The lettuce seedlings were stressed with 100 mg L^− 1^ of each (Cr, Cd, and Pb) and 100 mM NaCl.
Mycorrhizal and stressed (AMF + S)	The lettuce seedlings with AMF inoculum were stressed with 100 mg L^− 1^ of each (Cr, Cd, and Pb), and 100 mM NaCl.

### Measurements

#### Mycorrhizal colonization

To verify AMF colonization of the colonized plants at the end of the experiment (Thirty-two days after transplantation), lettuce roots were carefully cleaned with water to eliminate soil particles. The fine roots were chopped into 1-cm lengths and kept in a solution of formalin, acetic acid, and ethyl alcohol (5:5:90) (v/v/v). Root segments were then cleared with KOH (10%) at 90 °C for 7 min, suspended in 1 N HCl for 3 min with shaking, and dyed with trypan blue (0.05%)-lactophenol [30]. To ascertain the AMF colonization rates of the root, dyed segments were placed on glass slides and examined under a light microscope (10×). Fungal colonization and development (mycelium and vesicles) were seen in the inoculated lettuce roots. The mycorrhizal (MC), vesicles (VC), and arbuscules colonization (AC) percentages were calculated in the stained roots according to the Trouvelot et al. [31] approach.

#### Phenotypic parameters

To ascertain the impact of the combined NaCl and HMs stress application with or without AMF inoculation on lettuce morphology, root length (RL) (cm) was measured using a graduated ruler. The harvested plants’ shoot and root portions were weighed independently using an electric balance to record their shoot (SFW), besides root fresh weight (RFW) (g). The root and shoot portions were dried at 65 °C until their weights stabilized to estimate their shoot (SDW) in addition to root dry weight (RDW) (g). The root/ shoot (R/S) ratio as well as the number of leaves were recorded.

$$\frac{\mathrm{R}} {\mathrm{S}} \mathrm{ratio} \left( \% \right) = \frac{\left( \text{Root DW of plants} \right)} {\left( \text{Shoot DW of plants} \right)} \times 100$$

#### Physiological parameters

##### Photosynthetic pigments

The extracts of lettuce plant leaves (0.25 g) obtained by grinding with 10 mL of acetone (85%) were used in chlorophyll (Chl.) and carotenoid (Card) analyses [32] to show the alterations in their levels upon stress exposure. Using a UV–vis spectrophotometer (RIGOL-Model Ultra-3660), the absorbance was measured at 663, 644, and 452.5 nm against a blank of 85% acetone solvent. The Chl content was reported as mg g^− 1^ FW material.

$$\begin{aligned} \text{Chl a}\left(\text{ mg/g FW} \right) &= [12.7\mathrm{x} \left( \mathrm{A663} \right) \\&- 2.59\mathrm{x} \left( \text{A 644} \right)]\mathrm{x}\; \mathrm{V}/ \left( 1000\mathrm{x} \mathrm{FW} \right) \end{aligned}$$

$$\begin{aligned}\text{Chl b}\left(\text{ mg/g FW} \right) &= [22.9\mathrm{x} \left( \mathrm{A644} \right) \\&- 4.68\;\mathrm{x} \left( \text{A 663} \right)]\;\mathrm{x} \;\mathrm{V}/ \left( 1000\mathrm{x} \mathrm{FW} \right) \end{aligned}$$

$$\begin{aligned}\mathrm{Card}\left(\text{ mg/g FW} \right) &= \left(4.2\:\mathrm{OD}\:452.5 \right) \\&- \left(0.0264 \:\mathrm{Chl.a} + 0.426 \:\mathrm{Chl.b}\right) \\&\times \mathrm{E.V}/ \left( 1000\times \mathrm{FW}\right) \end{aligned}$$

* FW is the weight of the sample’s fresh material, and E.V. is the volume of the extracted solution.

##### Relative Water Content [RWC] and Water Saturation Deficit [WSD])

On the day of harvest, the leaf disc method was used to determine the RWC of the gathered leaf samples. First, fresh lettuce leaves were weighed and then steeped for four hours in deionized water. Following 48 h of drying at 65 °C in an oven, turgor weights (TW) and DW were measured [33]. Calculations were made using the formula given below:

$$\mathrm{RWC} \left( \% \right) = \frac{\left( \mathrm{FW} - \mathrm{DW} \right) } {\left( \mathrm{TW} - \mathrm{DW} \right) } \times 100$$

$$\mathrm{WSD} \left( \% \right) = 100 - \mathrm{RWC}$$

##### Membrane traits of lettuce leaves

To assess the membrane stability index (MSI) of lettuce leaves under both controlled and stressed environments, the Hayat et al. technique [34] was used. After being cut up, four leaves from each treatment were put in tubes with 30 mL of deionized water each. The tubes were incubated at 25 °C with shaking for 4 h before being measured for electrical conductivity (EC_1_), and then were autoclaved at 121 °C for 20 min, cooled to 25 °C, and their EC_2_ was assessed. Additionally, the ratio of the MSI of lettuce leaves under stress to that of the controls was used to assess membrane injury (MI) [35]. The EL, MSI, and MI were determined using the following formula: where MSIs refers to MSI of the stressed lettuce plants, and MSIc refers to MSI of the control ones.

$$\mathrm{EL} = [1 - \left( \mathrm{EC}_1 \: /\mathrm{EC}_2 \right)] \times 100$$

$$\mathrm{MSI} = \left( \mathrm{EC}_1 \: /\mathrm{EC}_2 \right) \times 100$$

$$\mathrm{MI} \left( \% \right) = [ 1 - \left( \mathrm{MSIs} \: /\mathrm{MSIc} \right)] \times 100$$

#### Genomic assessment

##### Genome fingerprinting by ISSR and SCoT analysis

Genomic DNA was extracted from lettuce leaves using DNeasy^®^ kits QIAGEN (cat# 1014630), in accordance with the guidelines provided by the manufacturer. The isolated DNA was subjected to resolution via 1% agarose gel electrophoresis in a 1 × Tris-acetate-ethylenediaminetetraacetic acid (TAE) buffer, supplemented with 0.5 µg mL^− 1^ ethidium bromide, to assess the integrity of the DNA.

The polymerase chain (PCR) reaction was conducted utilizing a Biometra thermal cycler (Germany), employing the primers for ISSR and SCoT regions of the genome that are enumerated in Table 2, within a reaction volume of 25 µL. The PCR and its associated cycling protocols were conducted in accordance with the methodology outlined by Abdelhameed et al., [36]. The reaction was carried out in a 25 µl reaction volume using 1 × COSMO DNA polymerase buffer (Willowfort, cat # WF10202001), 10 µM of each corresponding primer, 1 U of COSMO DNA polymerase enzyme, and 2 µl of the isolated DNA (≈ 150–200 ng). The PCR cycling protocol was conducted as follows: an initial denaturation step at 95 °C for 5 min, followed by 37 cycles comprising denaturation at 95 °C for 1 min, annealing at 50 °C for 30 s, and extension at 72 °C for 2 min. A concluding extension at a temperature of 72 °C for a duration of 10 min was incorporated. A 1.2% (/v) agarose gel prepared with a 1 × TAE buffer, incorporating 0.5 µg mL^− 1^ of ethidium bromide, was utilized to separate the PCR products. The GeneRuler 100 bp DNA ladder, catalog number SM0241 from Thermo Scientific, was employed to ascertain the size of the amplified fragments. The genomic template stability (GTS) was calculated according to the following equation [37]: ${\%}\;\mathrm{of}\;\mathrm{GTS}=\left(1-\frac{a}{n}\right)\times{100}.$ Where “n” is the total number of bands in control samples, and “a” is the average number of changes in DNA profile for each treatment.

**Table 2:** List of selected ISSR and SCoT primers, including their codes, sequences, and the total number of amplified bands by each primer

No.	ISSR-Primers codes	Sequencing (5′-3′)	Total no. of markers	SCot-Primers codes	Sequencing (5′-3′)	Total no. of markers
1.	I-868	(GAA)_5_	-	SCoT 13	ACGACATGGCGACCATCG	6
2.	I-827	(AC)_8_G	-	SCoT 22	AACCATGGCTACCACCAC	5
3.	HB14	(GT)_6_CC	-	SCoT 26	ACCATGGCTACCACCGTC	9
4.	I-842	(GA)_8_CTG	7	SCoT 27	ACCATGGCTACCACCGTG	-
5.	I-844	(CT)_8_GC	7	SCoT 28	CCATGGCTACCACCGCCA	7
6.	I-891	ACTACGACT(TG)_5_T	8			
7.	ISSR-5	(ACG)_4_GAC	5			

##### Comet assay

The Single Cell Gel Electrophoresis assay (SCGE), known as the comet assay, was used to assess the degree of DNA damage that could be induced by the applied stress and the possible repair that occurs, possibly due to AMF inoculation. Lettuce roots subjected to various treatments were collected and subsequently dissected into small fragments on a slide utilizing a pointed razor blade. The dissected roots were immersed in 500 µL of saline phosphate buffer (pH 7.5) and subsequently subjected to centrifugation at 3000 rpm for 5 min at a temperature of 20 °C. The obtained supernatant was subjected to centrifugation at 14,000 rpm for a duration of 5 min at 4 °C to precipitate the intact nuclei. The isolated nuclei were combined with 0.5% normal-melting-point agarose (NMPA) and subsequently deposited onto the surface of a slide, which was then promptly covered with a coverslip to form a thin coating. Following a 15-minute period during which the slides were maintained on ice, the cover was subsequently removed. The nuclei were then subjected to lysis using a lysis solution composed of 3 M NaCl, 10 mM Tris, 100 mM Na-EDTA, and 4% sodium hydroxide, adjusted to a pH of 10.0. This lysis process was conducted over a duration of 24 h at 4 °C in a dark environment. A 1% solution of Triton X-100 was incorporated immediately before application. The slides were maintained at room temperature within the electrophoresis apparatus for a duration of twenty minutes, which was filled with a freshly prepared alkaline buffer at pH 13, consisting of 300 mM NaOH and 1 mM Na EDTA. Subsequently, the slides within the electrophoresis tank were subjected to an exposure duration of 30 min at a voltage of 24 V (0.74 V/cm) and a current of 300 mA. After being removed from the electrophoresis tank, the slides were set aside on a drain tray. Subsequent to three cycles of rinsing with neutralization buffer (400 mM Tris, pH 7.5), the slides were subjected to a staining procedure for a duration of 5 min using 80 µL of ethidium bromide at a concentration of 20 µg mL^− 1^ to facilitate visualization [38, 39]. To eliminate any residual dye, the slides were subsequently rinsed with ice-cold water. A fluorescent microscope (AXiostar plus-Carl ZEISS) with a magnification of 200×, in conjunction with an OPTIKA digital microscope camera, was employed for visualization purposes. The fluorescent LED lamp was equipped with an excitation filter operating within the wavelength range of 520 to 530 nanometers. The CometScore v1.5 software was employed to analyze between 50 and 150 nuclei during each iteration. Visualization and analysis took place at the Animal Reproduction Research Institute (ARRI) in Giza, Egypt.

#### Biochemical analyses

##### Malondialdehyde [MDA] and Hydrogen Peroxide [H_2_O_2_]

The thiobarbituric acid reaction was used to measure the cell damage index of lipid peroxidation of lettuce leaves in terms of MDA concentration (nmol g^− 1^ FW) following stress exposure [40]. H_2_O_2_ content of lettuce leaf samples (500 mg) was calculated according to the Loreto and Velikova [41] method at 390 nm. Following homogenization in 5 mL (0.1%, w/v) TCA and centrifugation for 20 min, 1 mL of 1 M potassium iodide (KI) as well as 0.5 mL of 10 mM potassium phosphate buffer (pH 7.0) were combined with the supernatant (0.5 mL).

##### Osmoregulatory and 1^ry^ metabolites quantification

Using glycine betaine (GB) as a standard, the colorimetric approach was used to measure the GB content of lyophilized fresh lettuce leaves [42]. The lyophilized tissue was combined with 1.5 mL of 2 N H_2_SO_4_, heated in a water bath for 10 min at 60 °C, and then combined with 50 µL of cold KI-I_2_. After 16 h of 0–4 °C storage, samples were centrifuged for 15 min, and then put for 1 h in ice. After collecting the supernatant, 1,2-Dichloroethane (4.5 mL) was added. The mixture was incubated at that point incubated for 2 h at room temperature; additionally, the absorbance at 365 nm using a spectrophotometer was measured. Proline content in lettuce fresh leaves was measured by using the [43] method. The absorbance of proline was measured at 510 nm using 1 mL of glacial acetic acid and 1 mL of ninhydrin reagent. For the 1^ry^ metabolites quantification, using the phenol sulphuric acid reagent, the amount of total soluble carbohydrates (Tsc) in the tissues of dried lettuce shoots was calculated [44]. Protein contents were measured in lettuce fresh leaves by using the Lowry et al. [45] method.

##### Non-enzymatic antioxidants

The methodology of Jindal and Singh [46] determined the total phenolic content (Tpc) in lettuce leaves after being subjected to methanol extraction (80%), and the resulting extracts were analyzed using the Folin–Ciocalteu method at 650 nm. The quantification of Tpc was expressed in mg of Gallic acid (GA) equivalents/g FW. The quantification of total flavonoid content (Tfc) was assessed at 510 nm by the AlCl_3_ colorimetric assay [47]. Total antioxidant capacity (TAC) in lettuce extracts was assessed by mixing a reagent solution containing sulfuric acid (0.6 M), sodium phosphate (28 mM), and ammonium molybdate (4 mM) [48]. The tubes were placed in a boiling water bath for 90 min, and then they were allowed to cool before the absorbance at 695 nm was measured. µg ascorbic acid equivalents/g FW was the unit of measurement for the reported TAC.

##### Enzymatic antioxidants and phosphatases enzyme activities

Enzymes were extracted from 1 g of lettuce leaf tissues at 4 °C using 5 mL of extraction solution that contained 50 mM K-phosphate buffer at pH 7.6-, and 0.1-mM disodium ethylenediaminetetraacetate (EDTA) to measure the activity of antioxidant enzymes. Following a 15-minute centrifugation at 6000 rpm, the supernatant was used for enzyme tests. Ascorbate consumption at 290 nm was used to measure ascorbate peroxidase (APX) activity [49]. The pyrogallol technique was used to measure the activity of polyphenol oxidase (PPO) at 420 nm [50]. Pyrogallol was used as the substrate to measure peroxidase (POD) at 470 nm [51]. Additionally, the enzyme activity was expressed as U/µg FW, and the activity of phenylalanine ammonia-lyase (PAL) was measured at 290 nm using Zucker [52] as modified by the McCallum and Walker protocol [53]. Using the Tabatabai and Bremner method [54] and the *p*-nitrophenyl phosphate disodium salt (*p*NPP) technique, the activity of enzymes (acid [Acp, pH 5.0] and alkaline phosphatase [Alp, pH 8.0]) in lettuce leaves was determined. The appropriate homogenizing buffers were used to incubate the supernatant with 5 mM *p*NPP as a substrate to produce *p*NP. The activity was measured at 410 nm using colorimetry and expressed as nmol of *p*NP released/min.

#### Glomalin-related soil proteins

Rhizosphere soil was gathered to quantify the amount of readily extractable (EE-GRSP) and total extracted glomalin-related soil proteins (TE-GRSP) [55] using the Bradford assay at 595 nm on a UV–vis spectrophotometer [56]. To create EE-GRSP, two g of soil samples were mixed with eight mL of 20 mM sodium citrate (pH 7.0), autoclaved for thirty minutes at 121 °C, and then centrifuged for fifteen minutes at 6000 rpm. Until it was analyzed, the supernatant was maintained at 4 °C. For the TE-GRSP extraction process, a 2 g soil sample was autoclaved for 60 min together with 8 mL of 50 mM sodium citrate (pH 8.0), and centrifuged.

#### Metal contents in plant tissue

To assess the levels of Na^+^, Cr, Pb, and Cd, the lettuce root and shoot samples from all treatments were taken from 5 randomly selected plants. At first, the harvested plant tissues were rinsed in deionized water, dried at 65 °C, and subsequently powdered to a fine residue. Following acid digestion in a mixture of H_2_SO_4_, HNO_3_, and HClO_4_ (3:2:1, v/v), the Agilent 4210 MP-AES (Microwave Plasma Atomic Emission Spectrometer, Agilent Inc.) at the Ecology Laboratory, Faculty of Science, Capital University was used to quantify the concentrations of Na^+^, Cr, Pb, and Cd.

To evaluate lettuce’s capacity to withstand combined treatment, the metal tolerance index (TI) was measured [57]. The translocation factor (TF) measures the plant’s capacity to transfer accumulated metal from its roots to its aerial portions [58]. The total uptake (TU) of HMs was calculated [59]. The following formulas were employed:

$$\mathrm{TI} \left( \% \right) = \left( \text{DW of treated plants} \right)/ \left( \text{DW of control plants} \right) \times 100$$

$$\mathrm{TF} = \mathrm{C}_\mathrm{shoot} / \mathrm{C}_\mathrm{root}$$

$$\mathrm{TU} = \left( \mathrm{C}_\mathrm{shoot} / \mathrm{C}_\mathrm{root} \right) \times \mathrm{DW}_\mathrm{plant}$$

* DW stands for the dry weight of lettuce’s above-ground tissues or roots. The concentrations (µg/g DW) in the shoot and root are denoted by Cshoot and Croot, respectively.

### Statistical analysis

The morphological, physiological, and biochemical parameters of lettuce under the combined salt and HMs stress were affected by AMF application, and the statistical analysis was performed using SPSS software (Version 16.0, SPSS Inc., Chicago, IL, USA) [60]. The design of the experiment was completely randomized. Each treatment was administered in five replicas. Each replication’s mean value was utilized for statistical analysis, and significant differences were ascertained using one-way analysis of variance (ANOVA). Mean separations were compared using Duncan’s multiple-range test (DMRT) at a significance threshold of *p* ≤ 0.05 and presented as arithmetic means ± standard errors. The graphical presentation was created using Microsoft Excel version 2010. The heat maps were visualized using the R Studio interface and R software version 4.2.1 [61].

## Results

### Impact of stress on mycorrhizal traits

The combined multiple HMs-saline stresses boost the mycorrhizal characteristics percentages of AMF-colonized lettuce roots (Table 3). The MC% of the AMF-colonized lettuce roots under controlled and stressful conditions was 92.30 and 100%, respectively. However, there were no indications of the MC% of the non-inoculated control and the combined stressed treatment. Under combined salt and HMs stress, MC, VC, and AC rates increased dramatically to 8.34, 40.40, and 19.99%, respectively, in comparison to the AMF-non-stressed roots. Additionally, microscopic examinations revealed that AMF had colonized every lettuce root sample. The cortical cells of lettuce plant roots in the control (non-inoculated) lettuce roots do not exhibit any colonization (Supplementary Fig. 1a), while others that were analyzed had AMF-specific structures like intracellular hyphae (IH) and vesicles (Vesi) (Supplementary Fig. 1b, c, and d).

**Table 3:** Impact of the combined HMs (100 mg L^− 1^ of Cr, Cd and Pb) and salt (100 mM NaCl) stress on the mycorrhizal features of lettuce (*L. sativa* L.) plant roots expressed by Mycorrhizal (MC), Vesicular (VC), and Arbuscular colonization (AC) percentages in the root system

Treatments	MC (%)	VC (%)	AC (%)
C	0c	0c	0c
AMF	92.3 ± 2.44b	38.5 ± 1.017b	76.9 ± 2.04b
S	0c	0c	0c
AMF + S	100 ± 0.0a	54.0 ± 1.42a	92.3 ± 2.44a

*Data represent the mean of 5 replicates with standard error. Different letters indicate significant differences among treatments using a one-way ANOVA followed by Duncan’s multiple range test (*p* < 0.05). C; refers to control, AMF; AMF-inoculated plants, S; stressed plants with 100 mg L^− 1^ of Cr, Cd, and Pb and 100 mM NaCl, and AMF + S to AMF-inoculated and stressed plants with a combination of 100 mg L^− 1^ of Cr, Cd, and Pb and 100 mM NaCl

### Morphological parameters

The combined stress had a significant (*p* < 0.05) inhibitory consequence on lettuce growth in contrast to the control plants (Fig. 1a). It significantly reduced RL by 10.75% compared to the control (Fig. 1b). In terms of FW and DW, the combined stress suppressed both of the aboveground (18.26 and 15.27%) and belowground (17.82 and 11.30%) biomass (Fig. 1c and d). Conversely, AMF application outperformed non-inoculated lettuce plants under stress via promoting biomass generation and extending shoots and leaf number. Under controlled conditions, AMF application significantly (*p* ≤ 0.05) increased the RFW and RDW by 20.15 and 45.56%, and the SFW and SDW by 11.34 and 23.54%, respectively, compared to the non-inoculated control. AMF application under stress dramatically dropped the negative effect on these growth metrics (12.97 and 24.04% for shoots; 37.73 and 48.24% for roots). Compared to the stressed-lettuce plants, the RL was augmented by 34.40% and 26.50% in mycorrhizal-inoculated plants under controlled and stressed conditions, respectively. Furthermore, another significant finding was that R/S ratios of AMF-inoculated lettuce plants were greater than those of non-AMF plants cultivated in both control and stressed soil (Fig. 1b).

**Figure:**
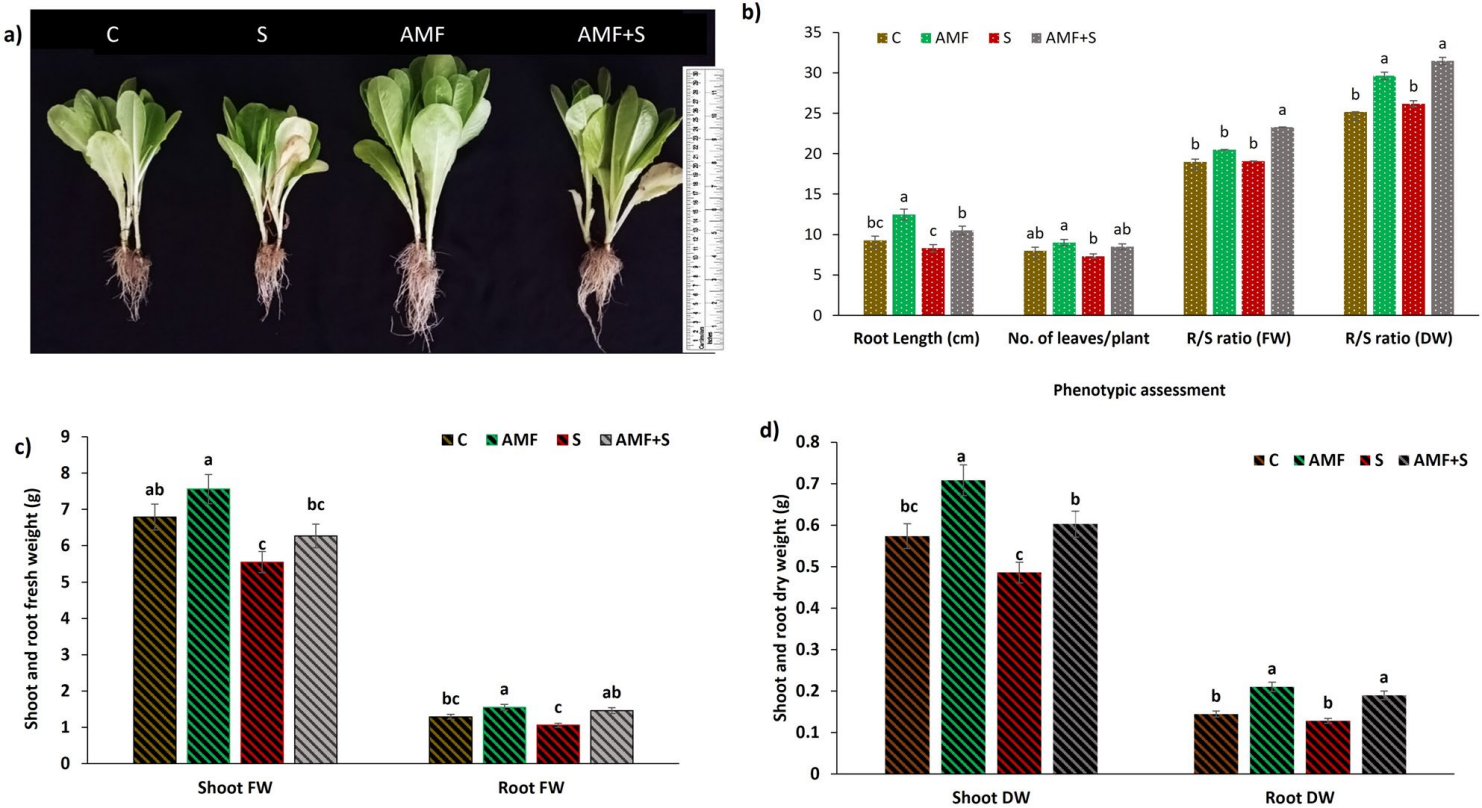
Effect of mycorrhizal colonization on the morphological appearance (shoots and roots) of 46-day-old lettuce (*L. sativa* L.) plants exposed to the combined HMs and salt stress conditions. **a **Qualitative phenotypic assessment. **b **Quantitative phenotypic assessment of root length, leaves number/plant, Root/shoot (R/S) ratio for both Fresh Weight (FW) and Dry Weight (DW). **c** Quantitative phenotypic assessment of shoot and root fresh weight (FW). **d **Quantitative phenotypic assessment of shoot and root dry weight (DW). *C; refers to control, AMF; AMF-inoculated plants, S; stressed plants with 100 mg L^− 1^ of Cr, Cd and Pb and 100 mM NaCl, and AMF + S; AMF-inoculated and stressed with a combination of 100 mg L^− 1^ of Cr, Cd and Pb and 100 mM NaCl

### Photosynthetic pigments

**Fig. 2:**
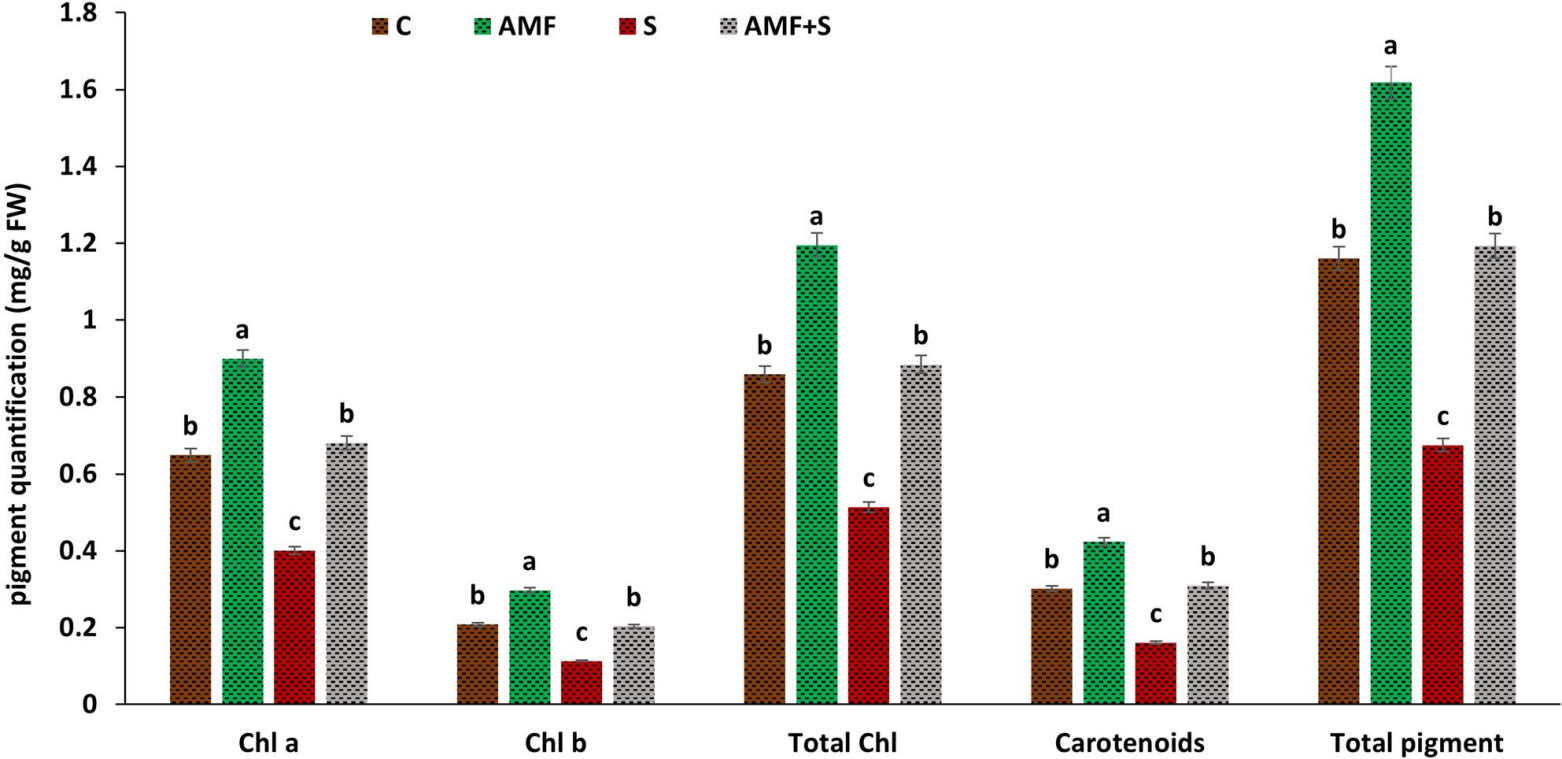
Impact of mycorrhizal colonization on pigment fractions (mg g^− 1^ FW) of lettuce (*L. sativa* L.) plant leaves exposed to the combined HMs and salt stress. *Values are means of 5 replicates with standard error. Different letters indicate significant differences among treatments using a one-way ANOVA followed by Duncan’s multiple range test (*p* < 0.05). C; refers to control, AMF; AMF-inoculated plants, S; stressed plants with 100 mg L^− 1^ of Cr, Cd, and Pb and 100 mM NaCl, and AMF + S; AMF-inoculated and stressed plants with a combination of 100 mg L^− 1^ of Cr, Cd, and Pb and 100 mM NaCl

The lettuce plants subjected to combined stress showed a significant (*p* < 0.05) reduction in all pigment contents compared with the controls. Stress exposure dramatically reduced Chl a, Chl b, Card, and TP by 38.21%, 45.67%, 46.68%, and 41.81%, respectively. Contrary to this, a proportional increase in their contents with AMF application, either under controlled or stressed conditions, was observed (Fig. 2).

### Water status and membrane stability

Under stressed conditions, AMF inoculation considerably elevated RWC and MSI compared with non-inoculated lettuce plants (Table 4). The percent of this elevation was 10.98% for RWC and 9.71% for MSI. Exposure of non-inoculated lettuce plants to combined HMs-saline stresses resulted in a notable decrease in RWC and MSI, with the lowest values recorded (18.74 and 12.59%). Concerning WSD, the results showed that lettuce leaves have a minimum WSD with AMF colonization under non-stressed (15.62 ± 0.413d) and maximum for those combined stressed (32.52 ± 0.860a). The MI decreased from 12.60 in non-AMF-inoculated lettuce plants to 4.11 in AMF-inoculated ones.

**Table 4:** Impact of mycorrhizal colonization on water status (RWC and WSD) and membrane traits (MSI, and MI) of lettuce (*L. sativa* L.) plant leaves exposed to the combined HMs (100 mg L^− 1^ of Cr, Cd, and Pb) and salt (100 mM NaCl) stress condition

Treatments	Relative water content (RWC) (%)	Water saturation deficient (WSD) (%)	Membrane stability index (MSI) (%)	Membrane injury (MI) (%)
C	81.09 ± 2.145a	18.90 ± 0.500c	87.86 ± 2.324a	----
AMF	84.37 ± 2.232a	15.62 ± 0.413d	88.95 ± 2.353a	----
S	65.89 ± 1.743c	32.52 ± 0.860a	76.79 ± 2.031b	12.60
AMF + S	73.13 ± 1.935b	25.91 ± 0.685b	84.25 ± 2.229a	4.11

*Data presented represent the mean of 5 replicates with standard error. Different letters indicate significant differences among treatments using a one-way ANOVA followed by Duncan’s multiple range test (*p* < 0.05). C refers to control, AMF AMF-inoculated plants, S Stressed plants with 100 mg L^− 1^ of Cr, Cd and Pb and 100 mM NaCl, and AMF + S; AMF-inoculated and stressed plants with a combination of 100 mg L^− 1^ of Cr, Cd, and Pb and 100 mM NaCl

### Genome profiling

**Fig. 3:**
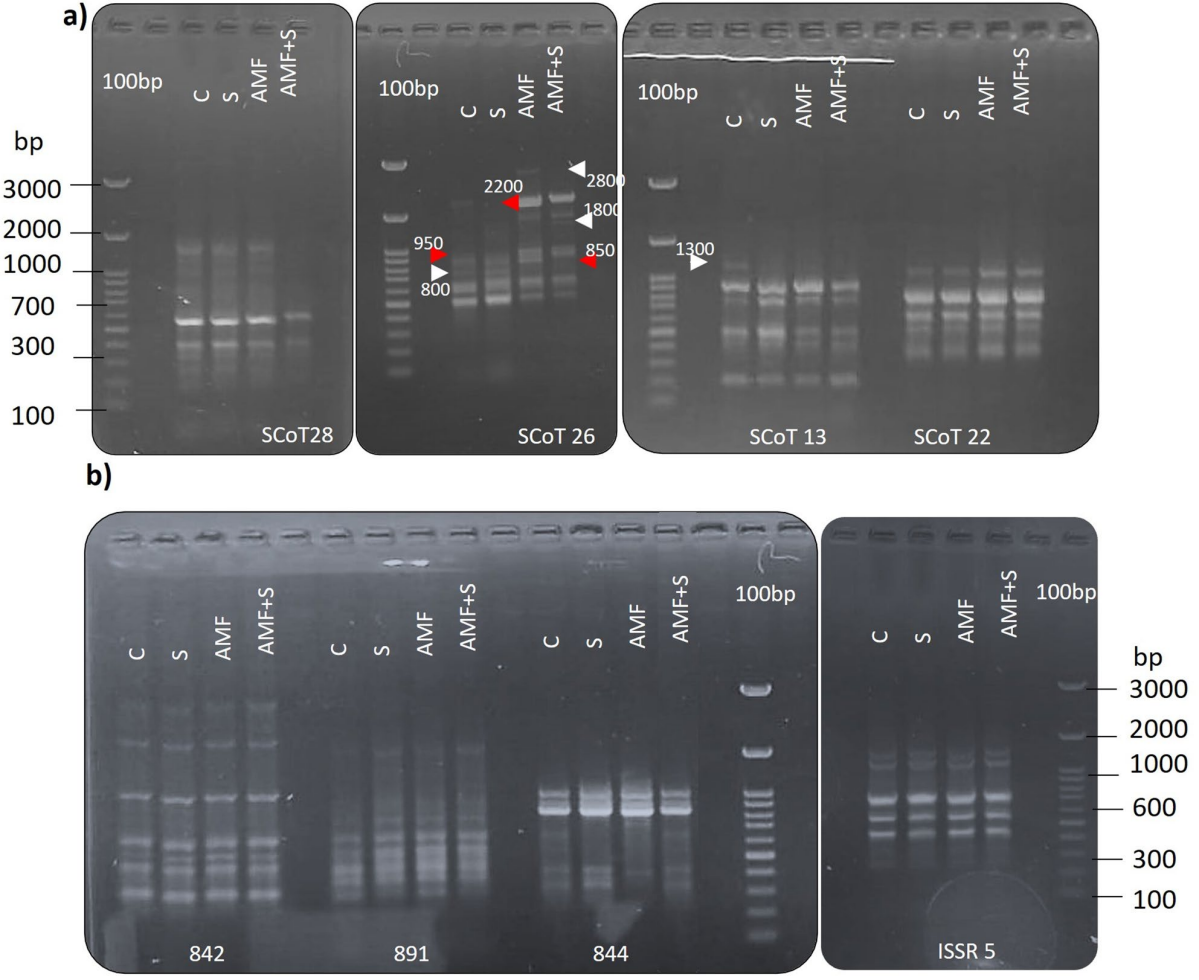
Effect of mycorrhizal colonization on the ISSR and Scot profiling of 46-day-old lettuce (*L. sativa* L.) plants exposed to the combined HMs and salt stress conditions. **a** SCoT profiling and (**b**) the ISSR profiling. The white arrows refer to band presence, and red arrows refer to band absence. C; refers to control, S; stressed plants with 100 mg L^− 1^ of Cr, Cd, and Pb with 100 mM NaCl. AMF; AMF-inoculated plants, and AMF + S; AMF-inoculated and stressed plants with a combination of 100 mg L^− 1^ of Cr, Cd, and Pb and 100 mM NaCl. 100 bp refers to the DNA ladder

A total of seven ISSR and five SCoT primers were employed to examine genomic fingerprinting in lettuce plants subjected to various treatments, including the combined stress and mycorrhiza inoculation. Only four ISSR and four SCoT primers successfully amplified consistent markers, as illustrated in Fig. 3. The amplified bands exhibited a size range from approximately 2800 bp to 200 bp. Notably, the ISSR primer I-891 yielded the highest number of bands, producing a total of 8 bands, whereas the SCoT 26 primer generated a maximum of 9 bands. In general, the ISSR analysis indicated a lack of variation among the various treatments, whereas some variations were noted in the SCoT analysis. The SCoT 26 analysis exhibited variability among the treatments, as the 800 bp band was notably absent in both mycorrhizal treatment groups (AMF and AMF + S), while the band of 950 bp was present only in these groups. A high molecular weight marker measuring 2800 bp was exclusively observed in the group inoculated with mycorrhiza. The band of 2200 bp was absent in the stress group, indicating the occurrence of DNA damage as a result of stress. The band measuring 850 bp was exclusively absent in the “AMF + S” group, whereas a narrow band measuring 1800 bp was uniquely present in this group. A band approximately 1300 bp in length for SCoT 13 was exclusively observed in the control group and was not present in any of the other treatments. The overall polymorphic bands (bands that differ among treatments) are 7. The control and AMF-inoculated plants exhibit four polymorphic bands, while the stress group has only two. The AMF + S treatment group retains an additional band to become three polymorphic bands. This observation was further corroborated by the calculation of genome template stability, which was reduced to 49% in the presence of stress, and increased to 74% when stress plants were colonized with AMF (Table 5).

**Table 5:** The Molecular Weights (Mwt) of polymorphic bands among various treatments as generated using SCoT primers (26 and 13). The presence (√) or absence (x) of each polymorphic marker band in each treatment group was denoted. The Genome Template Stability (GTS) was also calculated and expressed for each treatment group

Primer’s ID	Mwt of Polymorphic bands (bp)	C	AMF	S	AMF + S
SCoT 26	2800	X	√	X	x
	2200	√	√	X	√
	1800	X	X	X	√
	950	X	√	X	√
	850	√	√	√	X
	800	√	X	√	X
SCoT 13	1300	√	X	X	x
% GTS		100	100	49	74

*C refers to control, AMF AMF-inoculated plants, S Stressed plants with 100 mg L^− 1^ of Cr, Cd, and Pb and 100 mM NaCl, and AMF + S; AMF-inoculated and stressed plants with a combination of 100 mg L^− 1^ of Cr, Cd, and Pb and 100 mM NaCl

### DNA damage and genome integrity

**Fig. 4:**
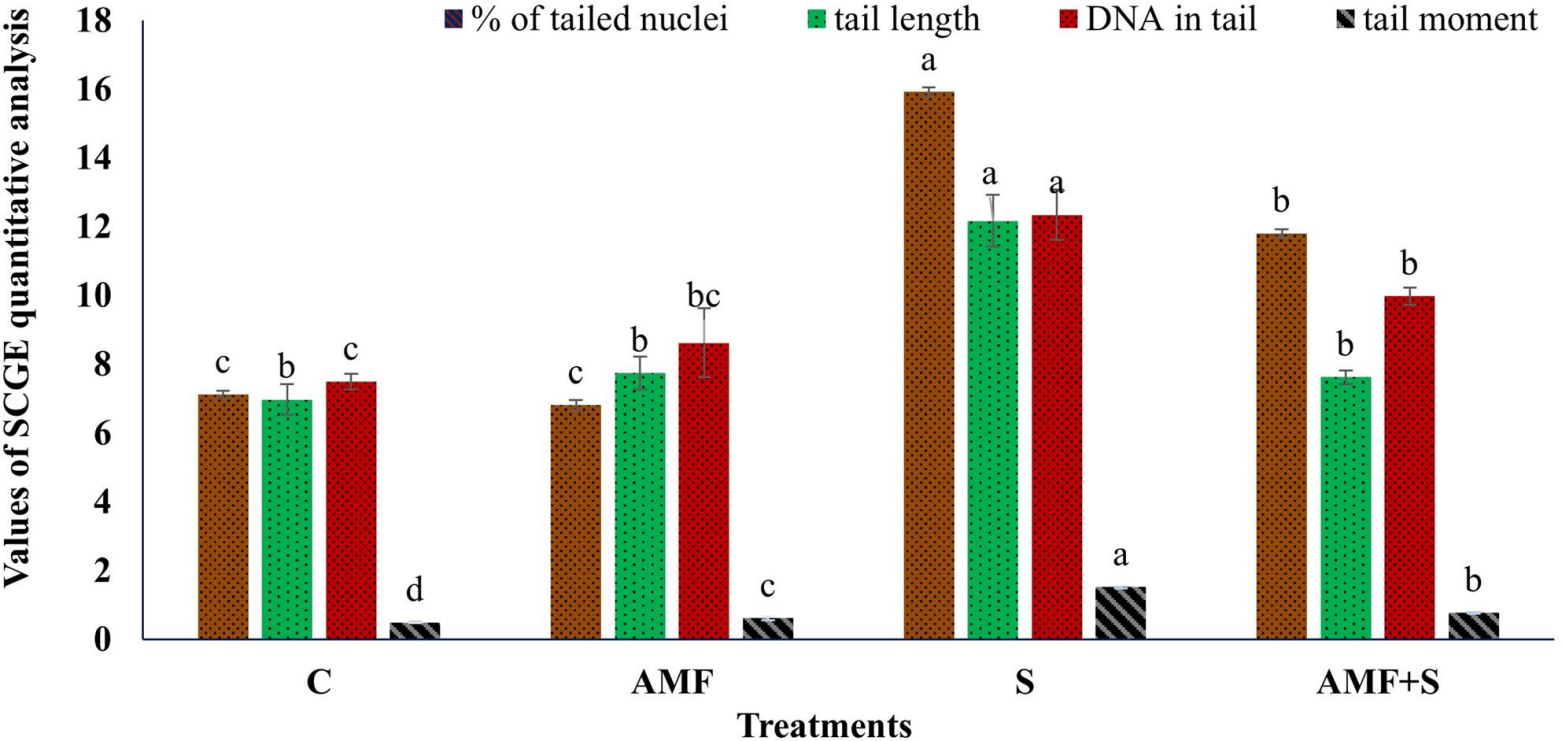
The quantitative results of Single Cell Gel Electrophoresis assay (SCGE) show the effect of mycorrhizal colonization on the DNA damage of 46-day-old lettuce (*L. sativa* L.) plants exposed to the combined HMs and salt stress conditions. C; refers to control, AMF; AMF-inoculated plants, S; stressed plants with 100 mg L^− 1^ of Cr, Cd, and Pb and 100 mM NaCl, and AMF + S; AMF-inoculated and stressed plants with a combination of 100 mg L^− 1^ of Cr, Cd, and Pb and 100 mM NaCl. Different letters indicate significant differences among treatments using a one-way ANOVA followed by Duncan’s multiple range test (*p* < 0.05)

The comet assay was utilized to determine the extent of DNA damage that could be caused by the imposed stress, as well as the potential for repair owing to mycorrhiza inoculation. Initially, the measured parameters of the mycorrhiza-inoculated plants did not exhibit any significant changes in comparison to those of the control plants (Fig. 4). The simultaneous exposure to the combined-HMs and salinity stress treatment may be associated with a significant increase in DNA damage, as suggested by the significant increase in all measured parameters of the comet assay. The significant increase was 2.24, 1.7, 1.65, and 3-fold more than the control group for the percent of tailed cells, tail length, DNA in tail, and tail moment. The inoculation of mycorrhiza substantially alleviates the damage resulting from the combined stress, as evidenced by a 25% reduction in tailed cells and a 48.8% decrease in tail moment when compared to the stressed plants (Fig. 4). This observation indicates the beneficial effect of mycorrhizal inoculation on lettuce under stress conditions by diminishing the DNA damage associated with such stress.

### Stress markers

**Fig. 5:**
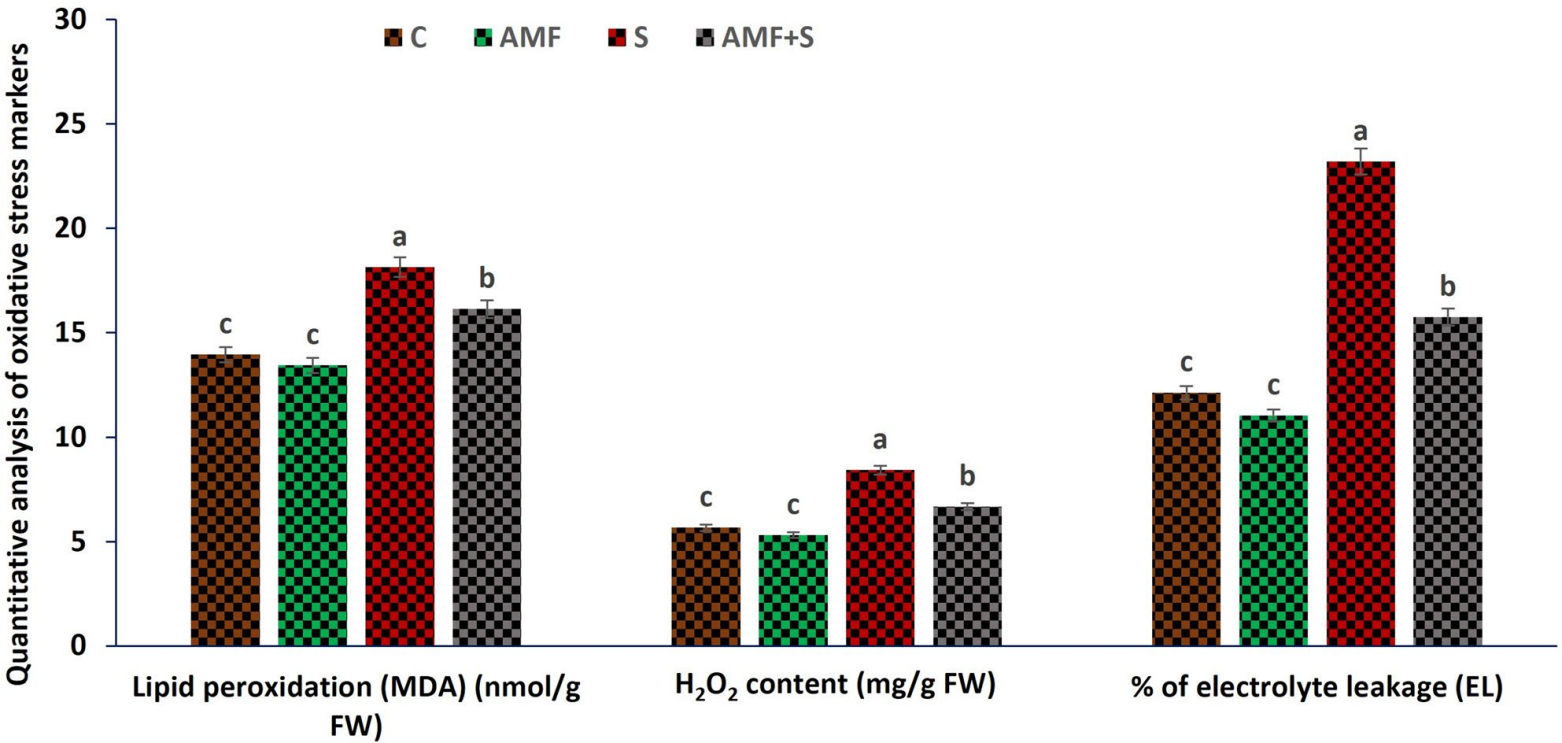
Impact of mycorrhizal colonization on the oxidative stress markers (lipid peroxidation [MDA] (nmol/g fwt), H_2_O_2_ content (mg/g FW)), and percent of electrolyte leakage (El) of lettuce (*L. sativa* L.) plants exposed to the combined HMs and salt stress conditions. *Data presented represent the mean of 5 replicates with standard error. Different letters indicate significant differences among treatments using a one-way ANOVA followed by Duncan’s multiple range test (*p* < 0.05). C; refers to control, AMF; AMF-inoculated plants, S; stressed plants with 100 mg L^− 1^ of Cr, Cd, and Pb and 100 mM NaCl, and AMF + S; AMF-inoculated and stressed plants with a combination of 100 mg L^− 1^ of Cr, Cd, and Pb and 100 mM NaCl

The combined HMs-salt stress led to destructive changes in cellular organelles associated with a substantial rise in ROS, especially H_2_O_2_, by (48.49%), the MDA content by (30.09%), and EL of the leaf membrane by (91.26%) compared to the control (Fig. 5). When comparing stressed lettuce plants colonized with AMF to those that were not, the levels of all oxidative stress indicators were significantly (*p* ≤ 0.05) reduced, by 11.03, 32.15, and 20.66%, respectively, providing cellular protection in comparison to those under stress.

### Osmoregulatory and 1^ry^ metabolites

**Fig. 6:**
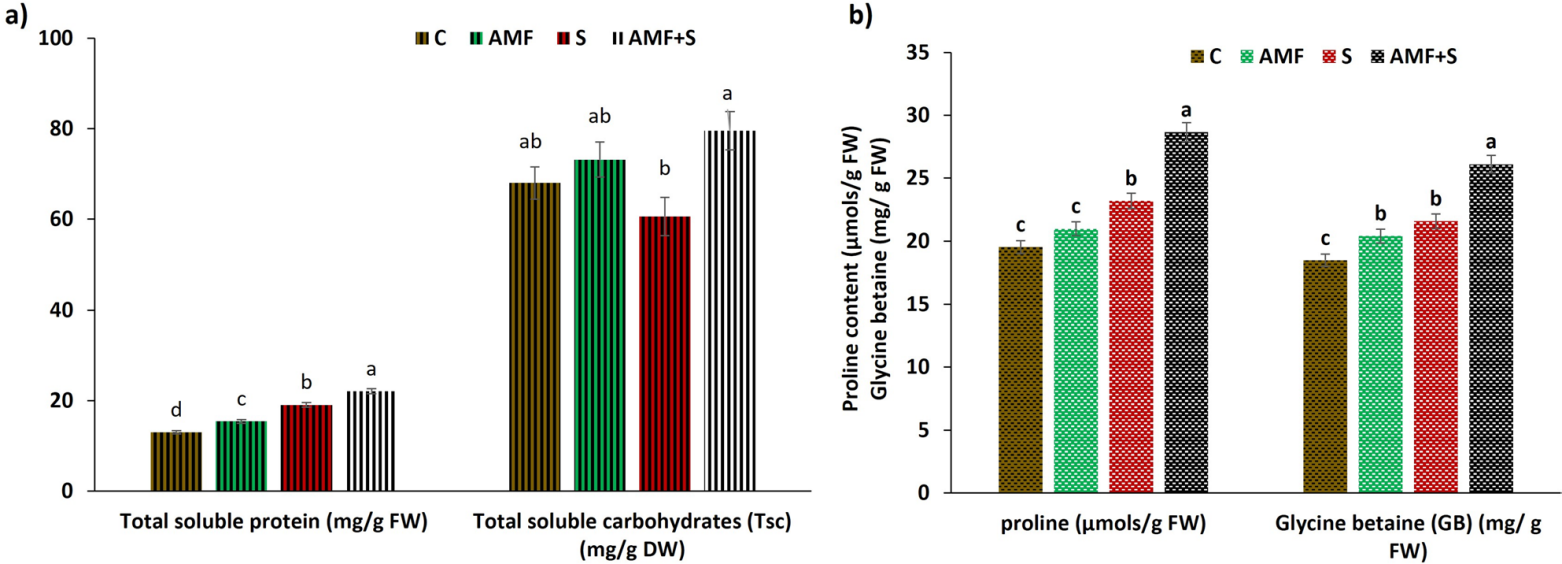
Impact of mycorrhizal colonization on the (**a**) 1^ry^ metabolites (total soluble protein and total soluble carbohydrates), and (**b**) osmoregulatory substances of lettuce (*L. sativa* L.) plant leaves grown under the combined HMs and salt stress. *Data presented represent the mean of 5 replicates with standard error. Different letters indicate significant differences among treatments using a one-way ANOVA followed by Duncan’s multiple range test (*p* < 0.05). C; refers to control, AMF; AMF-inoculated plants, S; stressed plants with 100 mg L^− 1^ of Cr, Cd and Pb and 100 mM NaCl, and AMF + S; AMF-inoculated and stressed plants with a combination of 100 mg L^− 1^ of Cr, Cd, and Pb and 100 mM NaCl

To assess whether AMF inoculation induces tolerance against HMs-salt stress in lettuce owing to non-enzymatic mechanisms, 1^ry^ metabolites represented by total soluble protein (Tsp) and carbohydrates (Tsc) were quantified (Fig. 6a). Upon stress, the AMF root colonization significantly (*p* ≤ 0.05) boosts the Tsp and Tsc by 15.7 and 31.1%, respectively, compared to the stressed samples. Similarly, mycorrhiza application upon exposure to stress was associated with a significant (*p* ≤ 0.05) upsurge of the osmoregulatory substances. Proline (23.27%) and GB (21.39%) contents significantly increased in AMF-colonized stressed plants compared to those under stress (Fig. 6b).

### Secondary metabolites and TAC

**Fig. 7:**
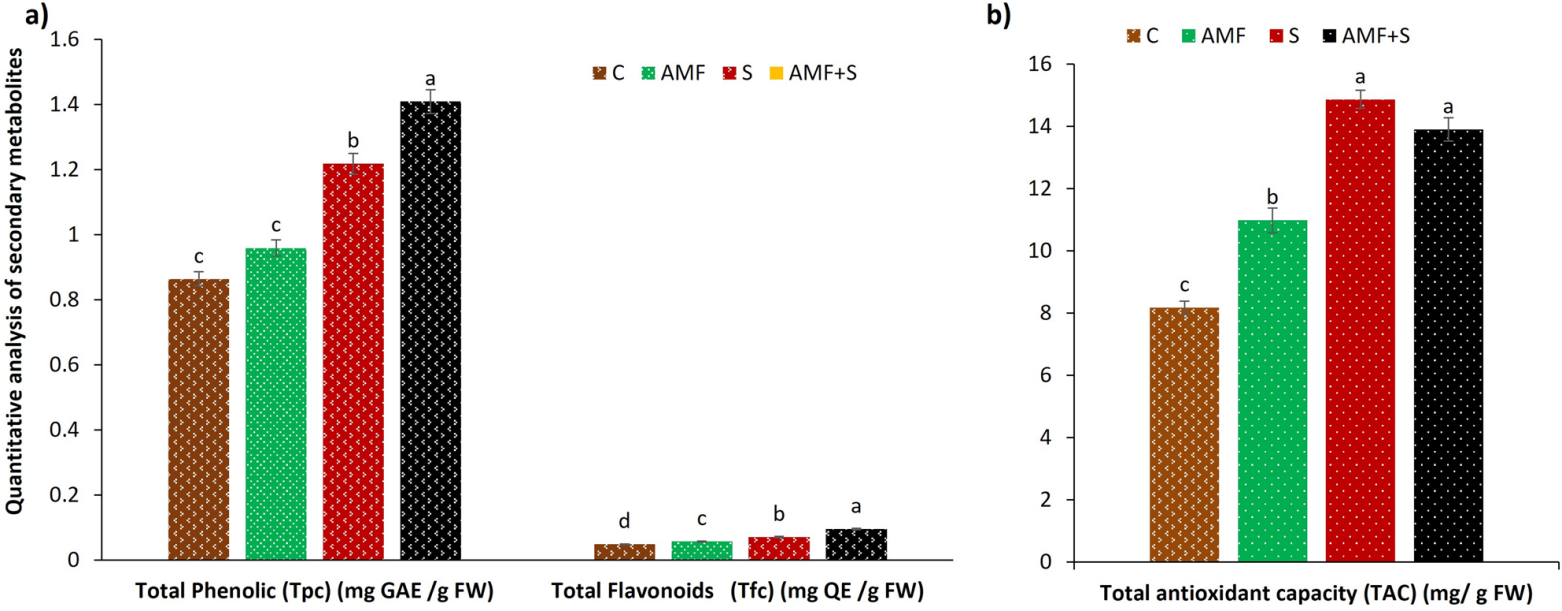
Impact of mycorrhizal colonization (AMF) on secondary metabolites of lettuce (*L. sativa* L.) leaves grown under stress conditions with a combination of 100 mg L^− 1^ of Cr, Cd, and Pb with 100 mM NaCl. **a** total phenolic (Tpc) (mg GAE /g FW) and total flavonoids (Tfc) (mg QE /g FW)], (**b**) TAC content (mg/g FW). *Data presented represent the mean of 5 replicates with standard error. Different letters indicate significant differences among treatments using a one-way ANOVA followed by Duncan’s multiple range test (*p* < 0.05). C; refers to control, AMF; AMF-inoculated plants, S; stressed plants with 100 mg L^− 1^ of Cr, Cd, and Pb and 100 mM NaCl, and AMF + S; AMF-inoculated and stressed plants with a combination of 100 mg L^− 1^ of Cr, Cd, and Pb and 100 mM NaCl

When compared to the control plants, stressed plants showed a considerable increase in the non-enzymatic antioxidants, such as Tpc and Tfc contents. As shown in Fig. 7a, the Tpc and Tfc of lettuce were measured as 0.864 mg GAE/g FW and 0.048 mg QE/ g FW in the control. However, under the combined stress, a significant (*p* ≤ 0.05) rise in their contents was observed (1.218 mg GAE/g FW and 0.071 mg QE/g FW**)**. The most substantial increase was observed in AMF-stressed plants. Under stress conditions, mycorrhiza colonization increased the Tpc (1.409 ± 0.037 mg GAE/g FW) and Tfc (0.095 ± 0.002 mg QE/ g FW) contents (Fig. 7a). A similar trend was reported for TAC. Under stress, lettuce leaves had the highest values of TAC (14.859 ± 0.393 mg/g FW) than those under controlled conditions (8.172 ± 0.216 mg/g FW). Mycorrhiza colonization in the presence of combined HMs and saline stress significantly (*p* ≤ 0.05) boosts TAC content, reaching (13.895 ± 0.367 mg/g FW) (Fig. 7b).

### Antioxidant enzyme system

**Fig. 8:**
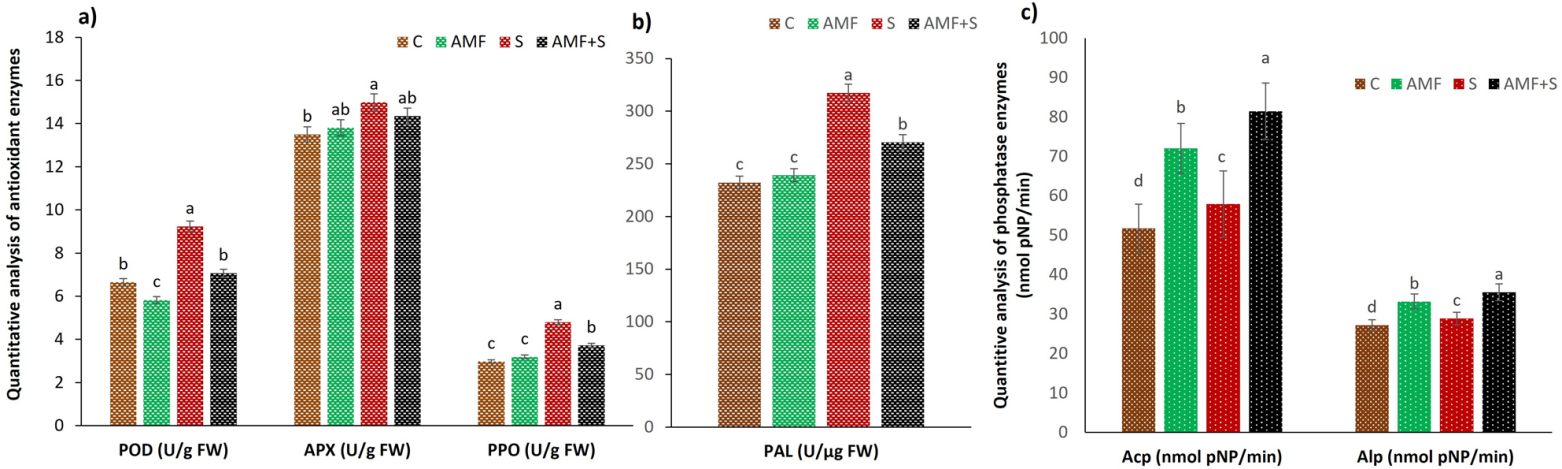
Impact of AMF colonization on antioxidant enzymes of lettuce leaves grown under a combination of 100 mg L^− 1^ of Cr, Cd, and Pb with 100 mM NaCl. (**a**) peroxidase **(**POD) (U/g FW), Ascorbate peroxidase (APX) (U/g FW), and Polyphenol oxidase (PPO) (U/g FW), (**b**) Phenylalanine ammonia-lyase) (PAL) (U/µg FW)], (**c**) acid phosphatases enzyme (Acp) (nmol pNP/min), and Alkaline phosphatases enzyme (Alp) (nmol pNP/min)]. *Data presented represent the mean of 5 replicates with standard error. Different letters indicate significant differences among treatments using a one-way ANOVA followed by Duncan’s multiple range test (*p* < 0.05). C; refers to control, AMF; AMF-inoculated plants, S; stressed plants with 100 mg L^− 1^ of Cr, Cd, and Pb and 100 mM NaCl, and AMF + S; AMF-inoculated and stressed plants with a combination of 100 mg L^− 1^ of Cr, Cd, and Pb and 100 mM NaCl

We investigated the activities of POD, PPO, and APX, and the findings showed that all measured enzymes significantly (*p* ≤ 0.05) increased as a result of the combined stress (Fig. 8a). Also, AMF inoculation contributed to a slight increase in APX and PPO. Under stress conditions, AMF application decreased the POD, APX, and PPO activities by 23.56, 4.28, and 22.38%, respectively, in contrast to non-AMF colonized plants. The PAL activity was significantly boosted upon stress (36.58%), while the AMF-colonization significantly reduced its activity by14.73% under stress (Fig. 8b). AMF application significantly enhanced the Acp activity to 72.05 ± 1.906 µmol *p*NP/min, and Alp to 33.179 ± 0.877 µmol *p*NP/min. The AMF colonization of combined HMs-salt stressed plants further boosted the Acp and Alp to 81.429 ± 2.154 µmol *p*NP/min and 35.554 ± 0.940 µmol *p*NP/min, respectively (Fig. 8c).

### Glomalin content

**Fig. 9:**
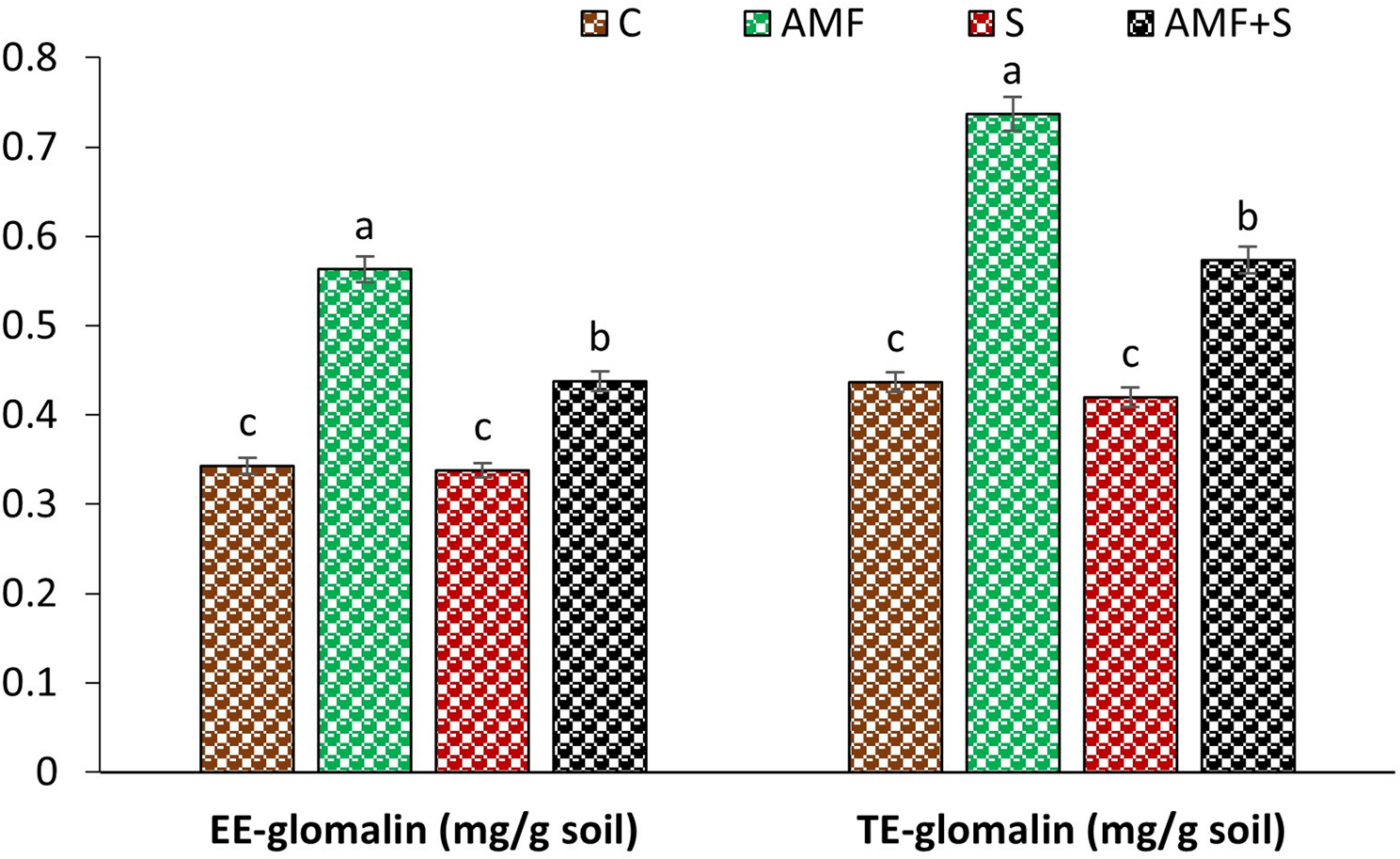
Impact of mycorrhizal colonization on easily extractable (EE-glomalin) and total extracted glomalin-related (TE-glomalin) soil proteins content (mg/g soil) in rhizospheric soils of lettuce (*L. sativa* L.) exposed to the combined HMs (100 mg L^− 1^ of Cr, Cd, and Pb) and salt (100 mM NaCl) stress condition. The data presented represent the mean of 5 replicates with standard error. Different letters indicate significant differences among treatments using a one-way ANOVA followed by Duncan’s multiple range test (*p* < 0.05). C; refers to control, AMF; AMF-inoculated plants, S; stressed plants with 100 mg L^− 1^ of Cr, Cd, and Pb and 100 mM NaCl, and AMF + S; AMF-inoculated and stressed plants with a combination of 100 mg L^− 1^ of Cr, Cd, and Pb and 100 mM NaCl

The present findings indicated that the AMF inoculation substantially increased the levels of T-GRSP and EE-GRSP in both control and HMs-salt-stressed plants (Fig. 9). The most substantial increase was observed for non-stressed AMF colonized plants. However, the AMF-colonized HMs-salt stressed plants showed 29.58% and 36.42% enhancement in EE-GRSP and TE-GRSP, respectively, compared to the non-AMF-colonized stressed plants. In contrast to the control, no substantial change in their contents was observed in response to the combined stress.

### Metals uptake and contents

**Fig. 10:**
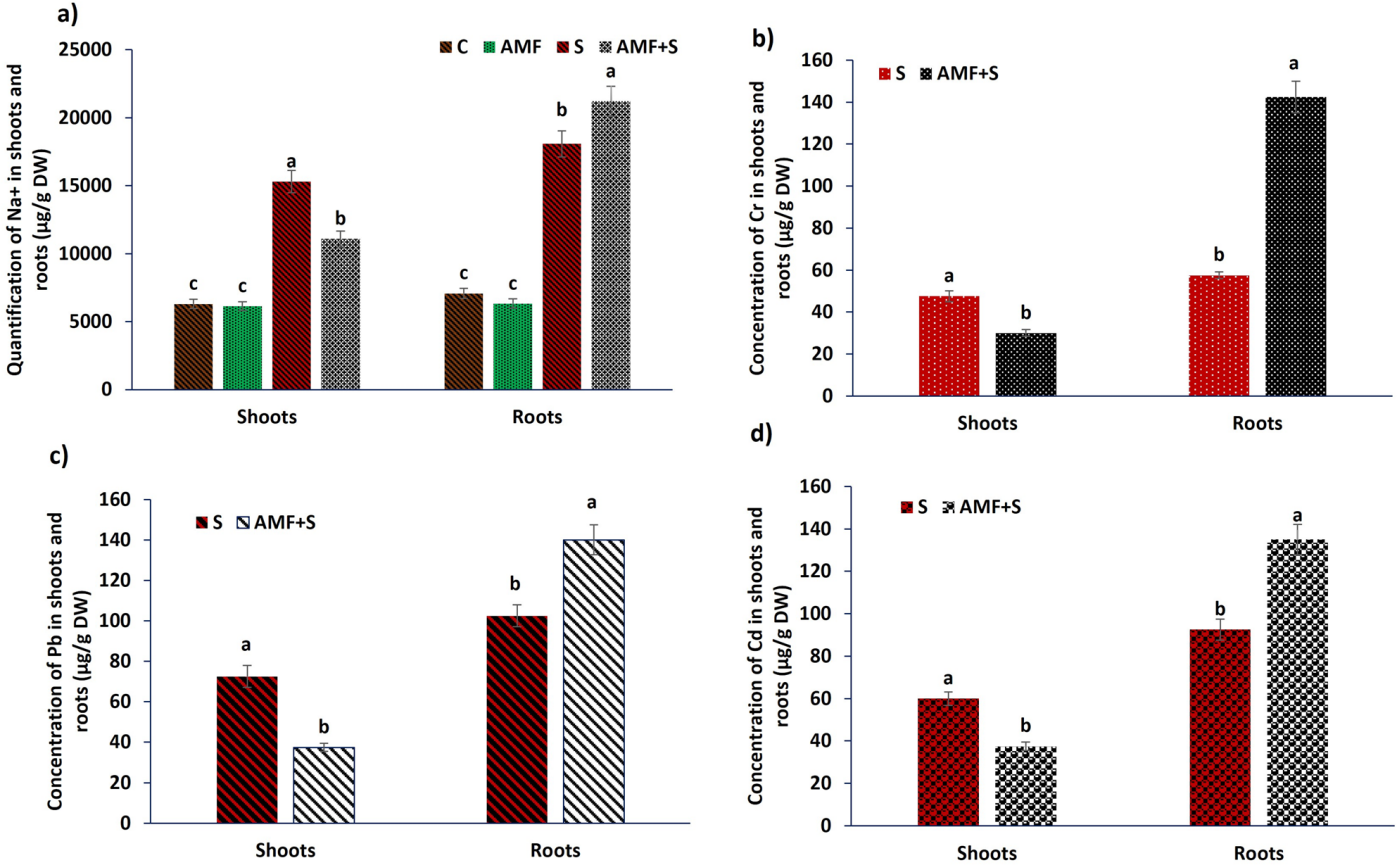
Impacts of mycorrhizal inoculation on Na⁺ (**a**), Cr (**b**), Pb (**c**), and Cd (**d**) contents in the shoots and roots of lettuce (*L. sativa* L.) plants exposed to the combined HMs (100 mg L^− 1^ of Cr, Cd, and Pb) and salt (100 mM NaCl) stress conditions. *Data presented represent the mean of 5 replicates with standard error. Different letters indicate significant differences among treatments using a one-way ANOVA followed by Duncan’s multiple range test (*p* < 0.05). C; refers to control, AMF; AMF-inoculated plants, S; stressed plants with 100 mg L^− 1^ of Cr, Cd, and Pb and 100 mM NaCl, and AMF + S; AMF-inoculated and stressed plants with a combination of 100 mg L^− 1^ of Cr, Cd, and Pb and 100 mM NaCl

In addition to Na^+^, all applied HMs (Cr, Pb, and Cd) were detected in roots as well as shoots of the treated plants; however, they were accumulated to higher levels in the roots. Interestingly, compared to non-inoculated controls, AMF-inoculated lettuce plants gathered substantially higher concentrations of Cr, Pb, and Cd in their roots, but their shoots had significantly (*p* ≤ 0.05) lower amounts of these elements. For example, AMF-HMs-salt stressed plants’ root tissues accumulated significant amounts of Cr and Cd (142.5 and 135.0 µg g^− 1^ DW), whereas the equivalent concentrations in the shoots dropped (30.0 and 37.0 µg g^− 1^ DW). Also, the concentration was of a HMs-specific variety, with the order of accumulation as Pb > Cd > Cr (Fig. 10). This was consistent with the order of metal uptake from the soil (Table 6). Although Cr exhibited the lowest metal uptake concentration, it exhibited the highest translocation factors (0.82), which underscored its high mobility within lettuce tissue. On the other hand, Cd had the lowest TF at 0.65. The TF for all HMs has been substantially reduced by the AMF inoculation, with a reduction of 74.4, 61.4, and 56.9% in the case of Cr, Pb, and Cd, respectively. Furthermore, the metal tolerance index has increased to 110.6 in the stressed AMF-inoculated plants, as opposed to 85.9% in stressed plants.

**Table 6:** Translocation Factor (TF), Metal Tolerance Index (TI), and Total Uptake (TU) of Cr, Pb, and Cd in lettuce (*L. sativa* L.) plants exposed to the combined HMs (100 mg L^− 1^ of Cr, Cd, and Pb) and salt (100 mM NaCl) stress condition

Treatments	TF			TI (%)	TU _Cr_	TU _Pb_	TU _Cd_
	Cr	Pb	Cd				
C	0c	0c	0c	100.00 ± 5.29bc	0c	0c	0c
AMF	0c	0c	0c	128.85 ± 6.81a	0c	0c	0c
S	0.82 ± 0.043a	0.70 ± 0.037a	0.65 ± 0.034a	85.94 ± 4.54c	171.12 ± 9.05b	285.20 ± 15.09a	248.53 ± 13.15a
AMF + S	0.21 ± 0.011b	0.27 ± 0.014b	0.28 ± 0.014b	110.59 ± 5.85ab	217.78 ± 11.52a	224.09 ± 11.85b	217.78 ± 11.52b

*C refers to control, AMF AMF-inoculated plants, S Stressed plants with 100 mg L^− 1^ of Cr, Cd, and Pb and 100 mM NaCl, and AMF + S; AMF-inoculated and stressed plants with a combination of 100 mg L^− 1^ of Cr, Cd, and Pb and 100 mM NaCl

### Inter-relationship among treatments and parameters

**Fig. 11:**
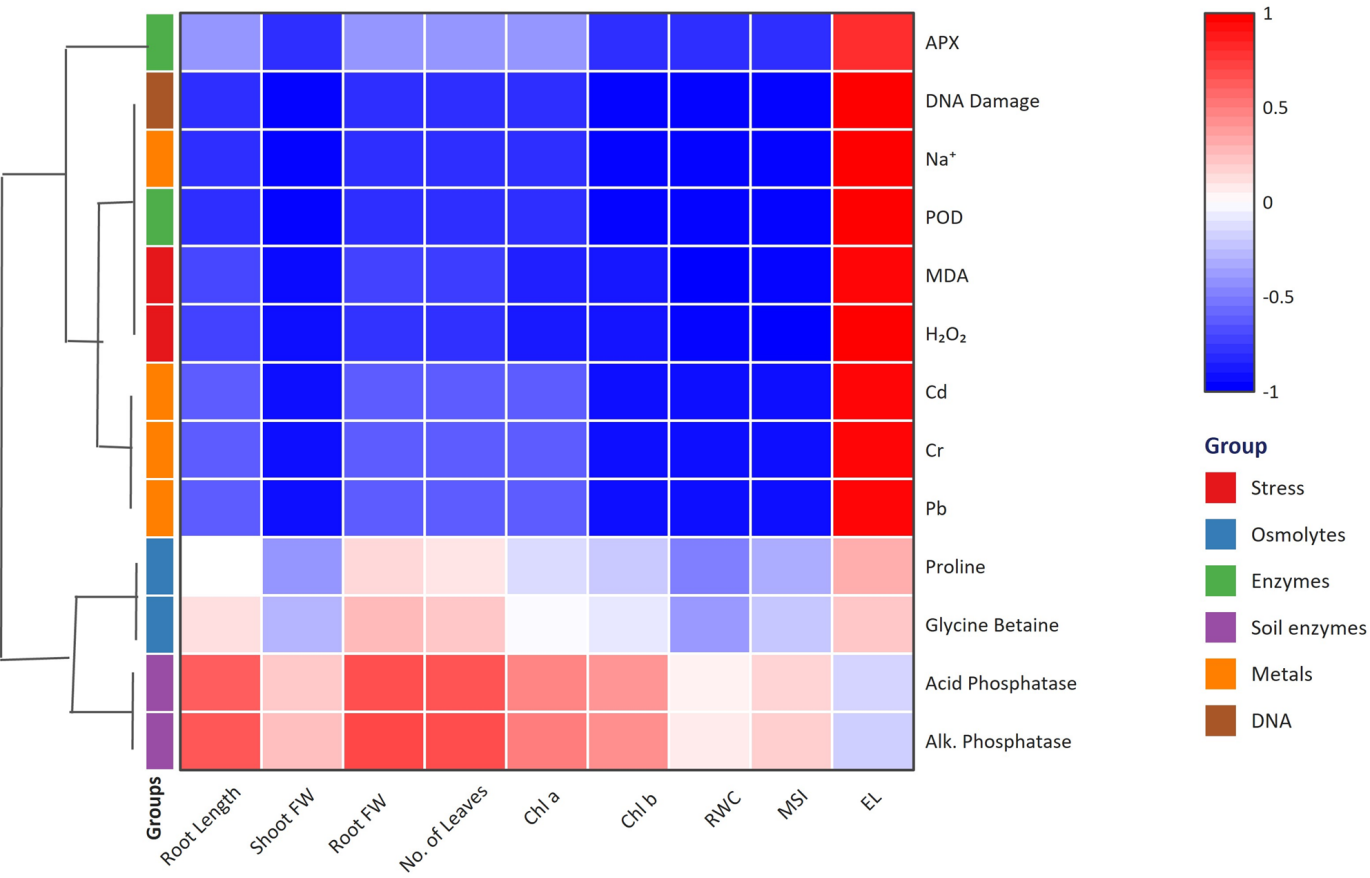
Hierarchical clustered heatmap illustrating the correlation patterns among physiological, biochemical, genetic, oxidative stress, antioxidant, and growth-related parameters under different treatments. Acp: acid phosphatase; Alp: alkaline phosphatase; APX: Ascorbate peroxidase; MDA: Malondialdehyde; EL: Electrolyte leakage; GB: Glycine betaine; MSI: membrane stability index; POD: Peroxidase; FW: Fresh weight; RWC: relative water content; H_2_O_2_: Hydrogen peroxide

**Fig. 12:**
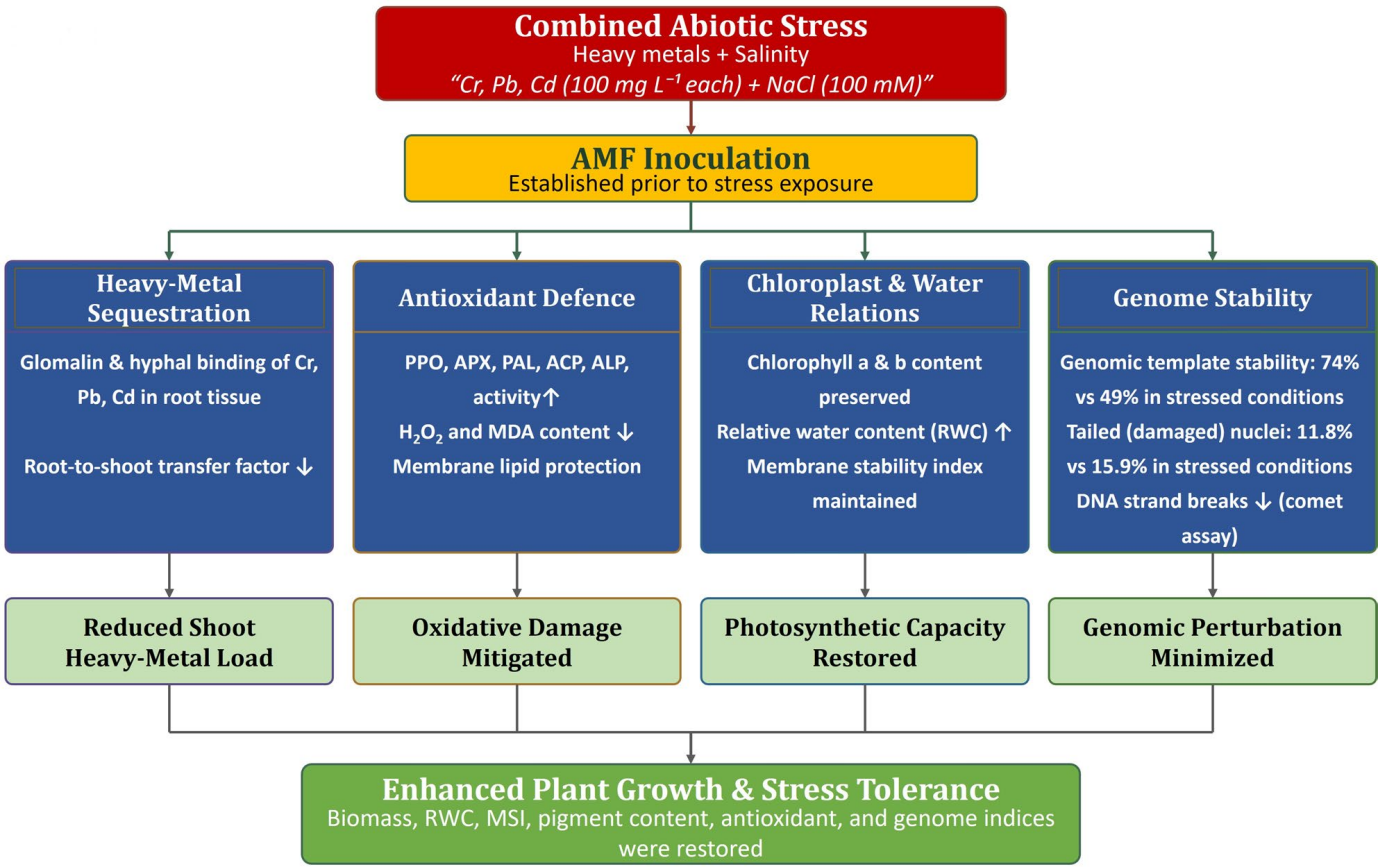
The mechanistic pathways for AMF-mediated protection against the combined phytotoxic impact of Cr, Pb, and Cd (each at 100 mg L^− 1^) with salt stress (100 mM NaCl). Four interconnected mechanisms are operating simultaneously. The first is HM sequestration. The second is antioxidant defense activation. The third is stabilization of chloroplast structure and water relations. The fourth is the mitigation of genomic perturbation

The grouped predictor–response heatmap highlights distinct functional clusters underlying plant stress adaptation (Fig. 11). Oxidative stress markers were negatively correlated with growth and photosynthetic traits, whereas osmoprotectants and antioxidant enzymes were positively associated with improved plant performance. HMs accumulation and DNA damage were consistently negatively correlated with physiological and growth-related variables, confirming their detrimental impact under combined stress conditions. The heatmap clustering revealed three biologically meaningful groups. The first and largest cluster comprised stress markers, antioxidant enzymes, metal content (Cd, Cr, Pb), Na^+^, and DNA damage, all of which were strongly negatively correlated with growth and physiological parameters but strongly positively correlated with EL. This pattern reflects a shared underlying mechanism, namely that HMs and salt accumulation drive oxidative stress and membrane destabilization, which in turn suppress growth and photosynthetic performance. The second cluster, comprising the osmolytes proline and GB, showed comparatively weaker correlations with growth traits, consistent with their role as compatible solutes that buffer osmotic stress rather than directly determine biomass accumulation. The third cluster, comprising the phosphatase enzymes (Acp, Alp), was positively correlated with root and leaf growth parameters but largely uncorrelated with stress and metal-related variables, reflecting their role in improving nutrient availability and root development independently of the direct stress response pathway. This clustering pattern supports the four interconnected protective mechanisms of AMF described in (Fig. 12) as HMs sequestration, antioxidant activation, chloroplast/water relations stabilization, and mitigation of genomic perturbation, which collectively explain why stress, metal, and DNA-damage parameters co-cluster and inversely track with growth and physiological recovery parameters.

## Discussion

HMs and salinity are abiotic stressors that impede plant growth by negatively affecting fundamental biological processes, such as photosynthesis, cell division, and overall development [62–64]. Therefore, there is an imperative need for environmentally friendly management techniques, i.e., applying AMF to boost crop output. It is extensively acknowledged that AMF inoculation provides plants with resistance to a variety of stressful environments by facilitating a series of complex mechanisms that result in an increased photosynthetic rate and water uptake, provide host plants with essential inorganic nutrients, and improve growth (as bio-fertilizers) [65, 66].

Our results showed that the combined stress boosts the mycorrhizal characteristics percentages of AMF-colonized lettuce roots. Under combined stress, MC, VC, and AC rates augmented dramatically (8.34, 40.40, and 19.99%, respectively) in comparison to those of the non-stressed ones. According to Riaz et al. [24], the existence of AMF in polluted soils may be connected to their ability to adapt to metal contamination. In contrast to our findings, Szada-Borzyszkowska et al. [67] who reported a 74% reduction in overall AMF colonization in *Miscanthus* × *giganteus* roots in HMs-contaminated soils after two growing seasons, and with reports of reduced colonization under NaCl and Cd stress in fenugreek [68, 69]. Such discrepancies may reflect species-specific differences in host–fungal compatibility and the magnitude of stress imposed.

The morphological characteristics of lettuce, including SFW, RFW, SDW, RDW, RL, and leaf number, are adversely affected by the combined salt and HMs stress. *Capsicum annuum* L. plants exhibited comparable outcomes when subjected to a combination of Cd and Pb stress [70], as well as to individual HMs stress with Cr in fenugreek [26] and Pb in poplar trees [71]. AMF inoculation, however, substantially improved these parameters, an effect attributable to enhanced concentrations of photosynthetic pigments and the consequent stimulation of photosynthetic activity (see Fig. 2) [26]. By extending the root’s absorptive surface through extraradical hyphae, AMF facilitate improved root-to-shoot ratios, broader soil nutrient access, and reduced metal toxicity [65, 71]. Combined HMs and NaCl stress significantly reduced photosynthetic pigment concentrations (Chl a, Chl b, carotenoids, Chl a + b, and total pigments) in lettuce leaves, likely due to thylakoid structural disruption, chloroplast membrane damage [71], stomatal closure, which alters membrane architecture, and reduces the intercellular levels of CO_2_ in chloroplasts [72]. AMF inoculation mitigated these effects by fortifying the photosynthetic machinery and enhancing resistance to Na⁺, Cr, Pb, and Cd toxicity [26, 66, 73], including through increased Mg uptake, improved stomatal conductance, and preserving Chl content in stressful situations [68].

The impaired reduction in the RWC of stressed lettuce leaves (65.89 ± 1.743c) may result from fluctuations in the concentrations of Na^+^ and Cl^−^, which disrupted root membrane permeability and reduced soil water potential [72]. This impairs cell development and division by decreasing turgor pressure and limiting water absorption [74].

AMF inoculation restored RWC (73.13 ± 1.935%) through expansion of the root surface area [75]. Correspondingly, AMF treatment improved membrane stability index (MSI; 84.25% vs. 76.79% in stressed non-inoculated plants) and reduced electrolyte leakage (EL) and membrane injury (MI). These improvements reflect AMF’s capacity to counteract the disruption of lipid and protein membrane components induced by HMs and salt exposure [76], and to eliminate reactive oxygen species (ROS) that underlie oxidative damage and lipid peroxidation.

Comprehending the alterations that transpire at the genetic level will facilitate the formulation of strategies aimed at sustaining plant growth amidst concurrent environmental stresses. Exposure to HMs may lead to genetic damage via inducing double-strand breaks (DSBs) and by constraining essential proteins involved in various DNA repair mechanisms [77]. In the present study, The ISSR fingerprinting revealed no detectable band alterations, while SCoT revealed some changes across various treatments, suggesting the occurrence of some stress-associated genomic modifications (Fig. 3). It should be acknowledged, however, that both ISSR and SCoT are PCR-based, low-resolution marker systems and, as such, provide only indirect, qualitative indicators of genomic changes rather than direct evidence of genome integrity or specific DNA repair activity. The ISSR targets the inter-sequence simple repeats by means of the abundant microsatellite regions as binding sites, while SCoT targets regions adjacent to the start codon of genes. Consequently, SCoT is capable of identifying genetic variations within a particular gene that are associated with a specific trait, and the assessment of differential gene expression [78]. Both ISSR and SCoT markers have been utilized in the genetic diversity analysis, the examination of interspecific and generic genetic relationships, and the evaluation of genetic fidelity in plants [36, 62, 78–80]. Genotoxicity in *Salvadora persica* callus exposed to ZnO, SiO₂, and Fe₃O₄ nanoparticles was confirmed by ISSR [37], and minor ISSR-based genomic alteration alongside chromosomal aberrations was reported in *Vicia faba* under Cr stress [62]. The current study revealed that the genome template stability was reduced to 49% under the combined saline-HMs stress (Table 5), suggesting a degree of stress-associated genomic perturbation. This reduction may be partly associated with the oxidative stress induced by saline-HMs exposure [26, 63, 81], though GTS values should be interpreted with caution, given the inherent resolution limitations of dominant marker techniques. Individual exposure to HMs (Cu, Cr, Pb, Cd, and Zn) has been documented to yield fewer effects than their cumulative exposure under controlled laboratory conditions, with their effects being contingent upon both the dosage and the specific type of metal involved [82].

To complement and validate these marker-based observations, the comet assay was employed as a more direct measure of DNA strand integrity. The comet assay confirmed a significant increase in DNA breaks under stress conditions, as reflected by elevated percentages of tailed cells and tail moment values in root cells of stressed plants (Fig. 4), consistent with reported findings in *A. cepa* under similar multi-metal exposure [82]. Importantly, AMF-inoculated plants subjected to the same stress maintained a GTS of 74% compared to 49% in non-inoculated stressed plants, and showed a reduction in tailed cells from 15.9% to 11.8% as evidenced by the comet assay. Taken together, these complementary lines of evidence, marker-based profiling and direct DNA damage quantification, suggest that AMF inoculation may contribute to mitigating stress-induced genomic perturbation. This confirms AMF’s ability to protect genome integrity and DNA repair systems. Though the precise molecular mechanisms underlying this effect warrant further investigation. This protective effect may be partly attributed to the capacity of mycorrhizae to reduce HMs translocation factors (Table 6), thereby limiting the exposure of nuclear DNA to metal-induced oxidative damage and supporting cellular homeostasis [83]. The interactions between metals may induce either an additive or a synergistic effect on genetic structures. DNA damage as well as programmed cell death (PCD) have similarly been documented in *V. faba* and *A. cepa* root cells after Ni and Cr exposure [84]. Previous studies have addressed the role of AMF under single-metal stress. Apodaca et al. established that AMF reduced Cu accumulation in spearmint roots while enhancing Mn, Zn, and Mg absorption, and improving antioxidant defenses [85]. The current study demonstrates for the first time that AMF can reduce genomic perturbation, as indicated by both molecular marker profiling and comet assay data under the combined challenge of HMs (Cd, Cr, and Pb) and salinity stress in lettuce.

The production of ROS, such as MDA and H_2_O_2_, in lettuce leaves is increased by the combination of stresses. Consistent with this, Pb significantly increased the levels of H_2_O_2_ and MDA in *Populus simonii* [71], and in fenugreek and tomato under individual salt, Cd, and Cr stressors [26, 69, 86]. AMF inoculation significantly reduced both H₂O₂ generation and MDA accumulation, demonstrating a protective role in preserving membrane integrity and mitigating salt- and HMs-induced oxidative stress and lipid peroxidation [66, 71].

Plants employ a diverse array of antioxidant defense mechanisms, such as enzymatic and non-enzymatic systems, to maintain cellular redox equilibrium [24]. In comparison to non-inoculated lettuce plants, our results indicated that AMF significantly elevated the levels of osmolytes (GB and proline) and 1^ry^ metabolites (proteins and Tsc) in both controlled and stressed lettuce plants. A high-water potential is maintained by high proline levels, which sustain Ca^+ 2^ accumulation and maintain membrane and protein structures [72]. Proline and sucrose accumulation have been shown to assist plants in withstanding Cr stress [62]. Total phenolic content (Tpc) and total flavonoid content (Tfc) also increased following AMF inoculation, indicating upregulation of the phenolic biosynthesis pathway and flavonoid-mediated adaptive response to salt stress [87]. These compounds contribute to ROS scavenging and serve as enzyme cofactors, regulating plant growth from embryogenesis to senescence [88].

Besides non-enzymatic antioxidants, enzymatic ones, PPO (4.788 ± 0.126 U/g FW), APX (14.980 ± 0.396 U/g FW), POD (9.235 ± 0.244 U g⁻¹ FW), and PAL (317.246 ± 8.393 U µg⁻¹ FW), were all significantly elevated under combined stress. PAL is a key enzyme in the phenylpropanoid pathway, catalyzing the deamination of L-phenylalanine to trans-cinnamic acid, and plays a central role in plant adaptation to abiotic stresses such as salinity, drought, and HMs toxicity [89]. PAL induction is frequently associated with increased Tpc and Tfc accumulation, thus preventing oxidative damage [90]. Although some antioxidant enzyme activities in AMF-inoculated stressed lettuce plants were lower than those observed in stressed non-inoculated plants, this reduction may indicate decreased oxidative pressure likely related to AMF-mediated stress alleviation rather than a weak defense response. Our results corroborate the findings of Shahvali et al. [91] who discovered that *Cucumis sativus* L. inoculated with *R. intraradices*, *Claroideoglomus etunicatum*, and *F. mosseae*, enriched growth, increased the amount of proline and phenol under salinity to detoxify ROS and reduce cellular damage.

Alp and Acp enzymes, which catalyze the hydrolysis of organic phosphates, releasing easily absorbed inorganic phosphate ions critical for plant growth and nutrient balance [92], were markedly increased by AMF inoculation under both controlled (39.20% and 22.07%, respectively) and stressed (40.56% and 23.84%) conditions. AMF not only extend the root’s absorptive surface through their extraradical hyphae but also stimulate the activity of phosphatases either directly by producing their enzymes or indirectly by enhancing the plant’s enzymatic response. Under abiotic stress, AMF colonization can mitigate damage by improving P acquisition through enhanced phosphatase activity [92].

Glomalin-related soil protein (GRSP), a heat shock protein or fungal glycoprotein that could bind and sequester HMs like Cu, Cr, Pb, Cd, and Zn, was produced by AMF and is indispensable for plant growth as well as soil health [93]. Combined stress reduced GRSP concentrations; however, AMF colonization significantly elevated EE-GRSP and TE-GRSP levels in the rhizospheric soil of lettuce plants, thereby improving soil quality and plant stress tolerance. The metal-binding functional groups of GRSP render it effective in forming complexes with HMs [94], stabilizing soil aggregates and facilitating plant access to deeper soil layers via the AMF hyphal network [95]. These findings support the role of GRSP in bio-stabilization of HMs [96].

The simultaneous application of salt and combined HMs may influence metal bioavailability through multiple ion–ligand interactions. The presence of Cl^−^ may enable the formation of stable organic ligands that modulate HMs toxicity [97]. On the flip side, Cr, Cd, and Ni impede the accumulation of Na in *Hordeum vulgare* L. and *Portulaca oleracea* L., whereas *P. crithmoides* L. and *Plantago coronopus* L. are not, indicating a plant-specific interaction between Na^+^ and HMs. At elevated salinity, an ionic exclusion mechanism may act indiscriminately on Na^+^, Cr, Cd, and Ni [98]. Under the combined stress of salt and HMs, AMF colonization notably altered metal distribution in lettuce tissues, limiting the upward transfer of metals to aerial tissues and promoting their accumulation in the root system. This pattern reflects multiple complementary mechanisms: enhanced chelation within root tissues, immobilization in the rhizosphere by fungal exudates (GRSP), and metal sequestration in fungal structures (e.g., arbuscules, vesicles, and extraradical hyphae) [93, 99, 100]. Lettuce is recognized as an efficient HMs accumulator with effective detoxification mechanisms, including metallothionein production [101–103]; AMF colonization further augments this capacity by activating phytochelatin and metallothionein synthesis genes and facilitating vacuolar sequestration [83, 100, 104]. AMF plants consequently retained a lower burden of HMs in their photosynthetic organs, consistent with their enhanced physiological performance under stress. These results demonstrate how important AMF is in reducing HMs stress through root metal compartmentalization, a characteristic with significant ecological and phytoremediation implications.

The current data suggested that AMF exerts its protective effect under combined HMs and salinity stress, through four interconnected mechanisms operating simultaneously (Fig. 12). The first is HMs sequestration; AMF hyphae and GRSP physically bind and immobilize HMs predominantly in the root zone, reducing their translocation to aerial tissues. This is reflected in consistently lower shoot-to-root transfer in AMF-inoculated plants. The second is antioxidant defense activation. AMF upregulates enzymatic antioxidants (POD, PPO, and APX), which collectively scavenge ROS generated under oxidative stress. The downstream result is a measurable reduction in H_2_O_2_ accumulation and MDA, protecting membrane integrity. The third is stabilization of chloroplast structure and water relations. By alleviating oxidative burden and improving water uptake through the extraradical mycelial network, AMF preserves Chl a and b content, RWC, and MSI, which collectively restore photosynthetic capacity. The fourth is the mitigation of genomic perturbation. With reduced HM translocation and lower oxidative stress reaching the nucleus, DNA strand breakage is diminished. This is evidenced by higher genome template stability (74% vs. 49% in stressed controls) and fewer tailed cells in the comet assay, supporting continued cell division and homeostasis. Together, these four mechanisms converge to produce measurable improvements in overall plant growth and resilience.

## Conclusions

Our results offer new insights into the responses of plants to the combined phytotoxic impact of Cr, Pb, and Cd with salinity on lettuce. In addition, it indicates that lettuce plants’ growth, photosynthetic pigments, and stress tolerance are considerably enhanced by AMF in response to this stress. AMF not only enhanced growth and physiological indicators but also activated antioxidant defense systems to mitigate oxidative damage, as evidenced by decreased DNA damage. Additionally, it reduced the translocation of HMs to lettuce’s aerial parts, demonstrating their efficacy in HMs sequestration. These suggest that AMF enhance the plant’s natural defenses as an environmentally friendly and sustainable approach in phytoremediation, particularly in high-salinity and HMs-contaminated areas. This approach increases plant resilience and provides a long-term solution for controlling HMs contamination, as well as re-vegetating salt-HMs contaminated soils to boost agricultural productivity and ensure food security.

### Limitations and future research perspectives

Our study has some limitations that should be considered, such as the use of a single AMF species instead of a mixed culture and a relatively short duration, despite the insightful data it provides. Furthermore, the results have not yet been verified in field settings. Consequently, research in the future should prioritize field trials to verify the efficacy of AMF inoculation and other management strategies in realistic soil conditions, in addition to variable soil conditions. By concentrating on transcriptomic analysis, we can enhance our understanding of the internal mechanistic pathway, which will facilitate the development of a more resilient plant in soils that are extensively contaminated.

## Supplementary Information

Supplementary Material 1.

## Data Availability

The relevant datasets supporting the results of this article are included within the article.

## Abbreviations

AC: Arbuscules colonization
Acp: Acid phosphatase
Alp: Alkaline phosphatase
AMF: Arbuscular mycorrhiza fungi
APX: Ascorbate peroxidase
EE-GRSP: Easily extractable glomalin-related soil proteins
EL: Electrolyte leakage
GB: Glycine betaine
MDA: Malondialdehyde
MSI: Membrane stability index
PAL: Phenylalanine ammonia-lyase
POD: Peroxidase
PPO: Polyphenol oxidase
MC: Mycorrhizal colonization
MI: Membrane injury
RL: Root length
Pnpp: P-nitrophenyl phosphate
ROS: Reactive oxygen species
SCoT: Start codon targeted
SFW: Shoot fresh weight
SDW: Shoot dry weight
RDW: Root dry weight
RFW: Root fresh weight
R/S: Root/shoot
SCGE: Single Cell Gel Electrophoresis assay
Tsc: Total soluble carbohydrates
TAC: Total antioxidant capacity
TE-GRSP: Total extracted glomalin-related soil proteins
VC: Vesicles colonization
RWC: Relative water content
WSD: Water saturation deficit
Tpc: Total phenolic content
Tfc: Total flavonoid content
