# Supplementary material for: Mycorrhizal symbiosis maintains DNA integrity and cellular homeostasis in lettuce under combined heavy metals and salt stress conditions
Source: BMC Plant Biol. 2026 Aug 8;26:1335. doi: 10.1186/s12870-026-09544-0 (PMC13452129; doi:10.1186/s12870-026-09544-0)
Supplement: Supplementary file 1 [file 12870_2026_9544_MOESM1_ESM.docx]

Supplementary figures:

| **a)**    **HC** | **b)**    **IH** |
| --- | --- |
| **c)**    **Vesi** | **d)**    **Vesi**  **IH** |

**Figure 1:** Mycorrhizal structures observed in lettuce (*L. sativa* L.) plant roots cultivated in soil stressed with HMs and NaCl. Control non-inoculated lettuce roots (**a**). Structures characteristic of AMF **(b, c, and d)** using following abbreviations: HC refers to “healthy cells (non-colonized root cells)”, Vesi (vesicle), IH (intercellular hyphae).
